# Structural and functional insights into the selective inhibition of mutant tau aggregation by purpurin and oleocanthal in frontotemporal dementia

**DOI:** 10.1002/pro.70240

**Published:** 2025-08-27

**Authors:** Alladi Charanraj Goud, Ihor Kozlov, Patricie Skoupilová, Lukáš Malina, Sudeep Roy, Viswanath Das

**Affiliations:** ^1^ Institute of Molecular and Translational Medicine, Faculty of Medicine and Dentistry Palacký University and University Hospital Olomouc Olomouc Czech Republic; ^2^ Department of Medical Biophysics, Faculty of Medicine and Dentistry Palacký University Olomouc Olomouc Czech Republic; ^3^ Department of Biomedical Engineering, Faculty of Electrical Engineering and Communication Brno University of Technology Brno Czech Republic; ^4^ Institute of Molecular and Translational Medicine, Czech Advanced Technologies and Research Institute Palacký University Olomouc Olomouc Czech Republic

**Keywords:** frontotemporal dementia, MAPT mutations, oleocanthal, purpurin, seeding competency, surface plasmon resonance, tau aggregation, tauopathies

## Abstract

Tau aggregation driven by microtubule‐associated protein tau (*MAPT*) mutations is central to frontotemporal dementia pathology, yet no disease‐modifying therapies effectively target mutant tau. Here, we identify purpurin (PUR) and oleocanthal (OLC) as selective inhibitors of mutant tau aggregation using peptide models spanning the R2R3 interface. Biophysical and cellular assays demonstrated that both compounds more effectively inhibit the aggregation of mutant tau peptides compared to wild‐type, with PUR preferentially targeting V287I and N279K variants, and OLC showing broader inhibitory activity. Surface plasmon resonance and docking analyses revealed more stable interactions and lower binding free energies with mutant tau, consistent with their enhanced inhibitory effects. Computational studies using monomeric and fibrillar tau structures supported the mutation‐specific binding profiles of PUR and OLC. Atomic force microscopy and confocal imaging confirmed reduced fibril formation, while post‐transduction treatment assays showed that both compounds significantly suppressed intracellular tau propagation. Additionally, OLC reduced tau phosphorylation and oligomerization in SY5Y‐TauP301L‐EGFP cells expressing mutant tau. These findings highlight the potential of PUR and OLC as structurally distinct, mutation‐targeted inhibitors of tau aggregation and propagation, providing a rationale for their further development as candidate therapeutics for frontotemporal dementia.

## 1 INTRODUCTION

Tauopathies are characterized by abnormal tau aggregation, leading to neuronal dysfunction and neurodegeneration. While tau aggregation is prominently associated with Alzheimer's disease (AD), primary tauopathies such as frontotemporal dementia (FTD) are associated with mutations in the microtubule‐associated protein tau (*MAPT*) gene (Bloom, 2014; Hutton et al., 1998; Spillantini & Goedert, 2013). Over 50 pathogenic *MAPT* mutations disrupt tau–microtubule interactions, enhance aggregation, and alter isoform ratios, exacerbating pathology in FTD (Strang et al., 2019; Young et al., 2021).

Several mutations within the microtubule‐binding repeat domain drive disease‐specific tau aggregation. For instance, the S320F mutation stabilizes hydrophobic interactions, exposing amyloidogenic motifs and enabling spontaneous fibril formation (Chen et al., 2023). Similarly, P301L, V287I, and N279K mutations significantly increase aggregation propensity (Gao et al., 2018; Strang et al., 2019; Sun et al., 2023). P301L destabilizes structures near the amyloid‐forming PHF6 (306VQIVYK311) motif in the R3 region, while V287I and N279K enhance fibrillization by weakening microtubule‐binding interactions (Chen et al., 2019; Hong et al., 1998; Spillantini et al., 1998; Spittaels et al., 1999).

Tau aggregation follows a nucleation‐elongation mechanism, with the R2 and R3 regions playing a critical role in stabilizing intermediates and forming larger fibrils. Structural studies have identified the PHF6 and PHF6* (275VQIINK280) motifs as key aggregation‐prone sequences in tau, emphasizing their role in disease pathology (Zhu & Qian, 2022). Mutations like P301L disrupt local conformational elements surrounding these motifs, promoting intermolecular interactions that enhance aggregation propensity (Chen et al., 2019). Given that several pathological mutations occur at the R2R3 interface, these regions represent crucial therapeutic targets (Chen et al., 2019, 2023; Zhu & Qian, 2022).

A major therapeutic challenge is identifying small molecules that selectively target aggregation‐prone motifs in tau, particularly those affected by *MAPT* mutations. While many tau aggregation inhibitors have been developed, most have been designed for wild‐type (WT) tau, with limited emphasis on mutation‐specific mechanisms (Soeda & Takashima, 2020). Several natural compounds, small‐molecule inhibitors, such as methylene blue derivatives (LMTX), polyphenolic compounds (epigallocatechin and curcumin), and phenothiazine derivatives, have been shown to interact with tau, modulating their aggregation properties (Monteiro et al., 2024), but their selectivity for *MAPT* mutations has not been well characterized. Two such compounds, purpurin (PUR) and oleocanthal (OLC), have previously been shown to inhibit tau aggregation (Li et al., 2009; Monti et al., 2011, 2012; Viswanathan et al., 2020), but these studies primarily investigated their effects on WT tau, with less clarity on their selectivity for *MAPT* mutants. While OLC has demonstrated inhibitory effects on P301L tau aggregation, its comparative activity against WT tau remains less defined (Li et al., 2009). Given the structural differences introduced by *MAPT* mutations and their role in disease‐specific tau aggregation, it is critical to determine whether these inhibitors exhibit mutation‐specific activity or if their effects are uniform across different tau variants.

To address this, we examined the PUR and OLC's binding interactions and inhibitory effects on both WT and mutant tau (P301L, V287I, and N279K) using tau peptide models that represent the R2R3 interface. Peptides spanning R2R3—including PHF6 and PHF6*—reliably reproduce full‐length tau aggregation and are widely used in tau research (Chen et al., 2019; Viswanathan et al., 2020). We assessed the potential of both compounds as tau aggregation and propagation modulators through surface plasmon resonance (SPR) analysis, biophysical assays, and cellular seeding models.

We demonstrate that PUR and OLC act as mutation‐selective inhibitors, preferentially targeting tau peptides modeling regions of pathogenic *MAPT* variants (P301L, V287I, and N279K). These compounds not only prevent fibrillization but also abolish the formation of seeding‐competent fibrils, a key driver of tau pathology in neurodegenerative disorders. This mechanistic specificity—combining mutation selectivity with inhibition of seeding capacity—distinguishes these compounds from previously studied tau aggregation inhibitors. Our findings highlight their therapeutic potential and suggest that mutation‐targeted approaches could be crucial for tauopathy intervention.

## 2 RESULTS

### 2.1 WT and mutant tau peptides show distinct aggregation kinetics and morphologically diverse aggregates

**FIGURE 1:**
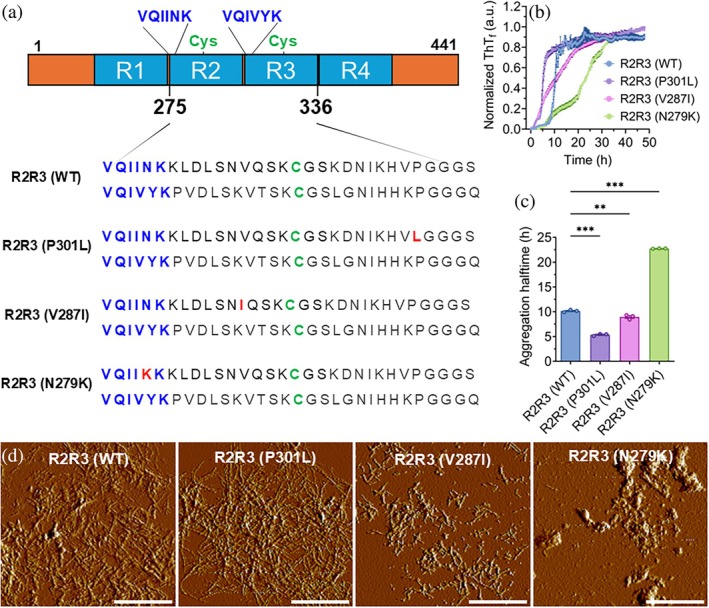
Peptides of tau form fibrillar and morphologically distinct aggregates. (a) Schematic representation of full‐length tau and amino acid sequences of tau peptides representing the R2R3 interface. Amyloid hexapeptide motifs are shown in blue, cysteine residues are marked in green, and mutations are indicated in red. (b) Aggregation kinetics of wild‐type (WT) and mutant (P301L, V287I, and N279K) R2R3 peptides at a concentration of 50 μM. Curves represent the mean of three replicates (individual replicate curves are shown in Figure S3). Source data are provided in the Data Availability Statement section. (c) Aggregation *t*_1/2_ derived from sigmoidal fitting of individual replicate curves (*n* = 3). Mean ± SEM. Individual replicate curves and corresponding sigmoidal fittings are shown in Figure S1. **p < 0.01, ***p < 0.001, one‐way ANOVA (Dunnett's multiple post hoc). (d) Morphology of aggregates formed by R2R3 peptides after 48 h of aggregation. Scale bar: 1 μm. ThT, thioflavin T.

To establish R2R3 tau‐derived peptides as reliable models for tau aggregation, we analyzed WT and mutant variants‐R2R3 (WT), R2R3 (P301L), R2R3 (V287I), and R2R3 (N279K) (Figure 1a)—using a thioflavin T (ThT)‐based aggregation assay. Prior to these assays, the monomeric state of peptides in aqueous solution was confirmed by ultraviolet–visible spectroscopy (UV–Vis) spectrometry and sodium dodecyl sulfate–polyacrylamide gel electrophoresis (SDS‐PAGE) under non‐reducing conditions (Figures S1 and S2). Free ThT is stable when it is free, but exhibits increased fluorescence upon binding to β‐sheet‐rich amyloid fibrils (Viswanathan et al., 2020), allowing real‐time monitoring of aggregation. Normalized ThT fluorescence curves were then fitted to a sigmoidal model to derive the aggregation halftimes (*t*_1/2_) as indicators of aggregation kinetics (Figure S3), which revealed distinct behaviors for WT and mutant tau peptides (Figure 1b). Mutant peptides P301L and V287I aggregated faster, with shorter *t*_1/2_ (5.37 and 8.94 h, respectively), while N279K peptide aggregated more slowly (*t*_1/2_: 22.74 h) compared to the WT peptide (*t*_1/2_: 10.15 h; Figure 1c).

Atomic force microscopy (AFM) revealed morphologically distinct aggregates formed by WT and mutant peptides after 48 h of aggregation (Figure 1d). The WT peptide predominantly formed long, unbranched fibrils characteristic of mature tau aggregates. The P301L peptide similarly formed abundant long fibrils, but these fibrils appeared more curved and bundled. The V287I peptide formed shorter, fragmented fibrils, while the N279K peptide produced amorphous aggregates, lacking ordered fibrillar structure.

### 2.2 Differential binding affinities and dynamics of PUR and OLC with WT and mutant tau peptides

**FIGURE 2:**
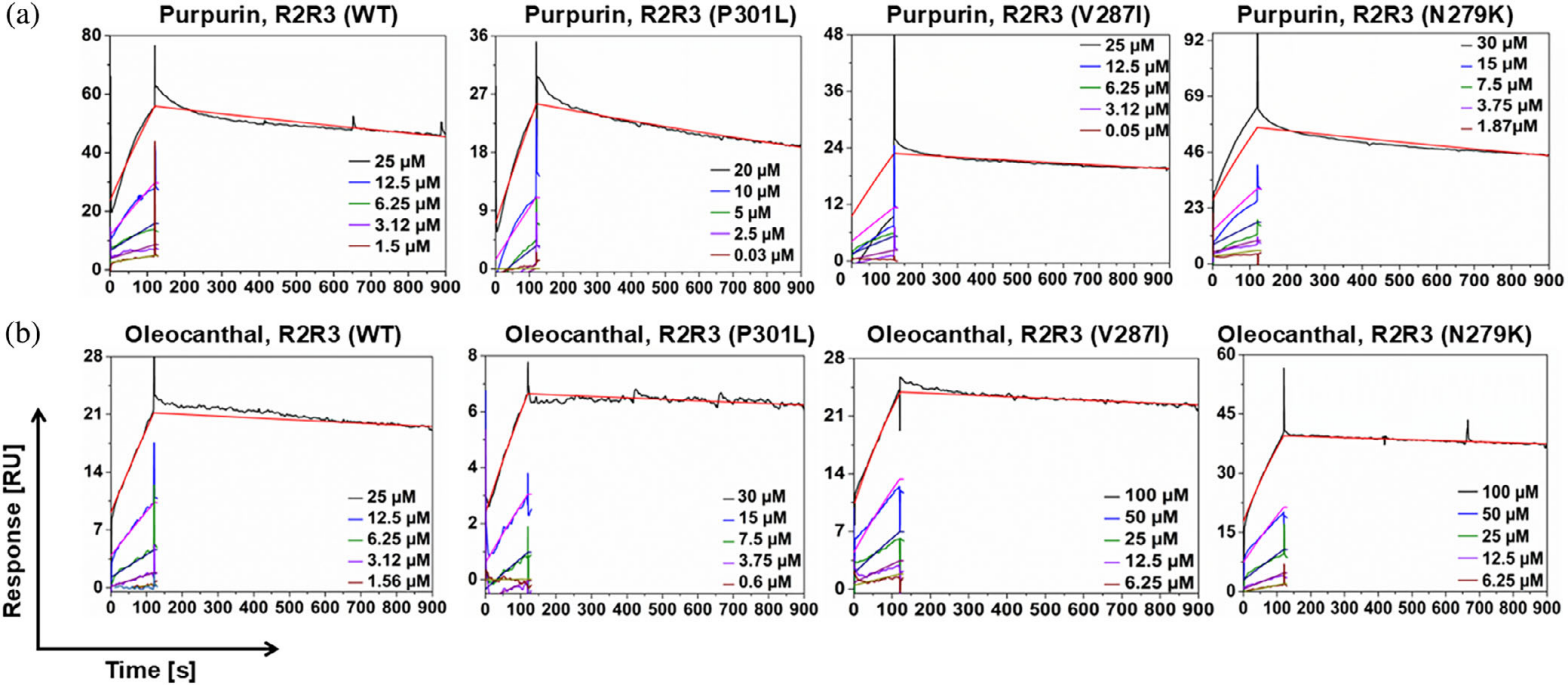
Titration cycle kinetics of purpurin (PUR) and oleocanthal (OLC) binding with wild‐type (WT) and mutant tau peptides. Surface plasmon resonance (SPR) sensorgrams showing the binding interactions of (a) PUR and (b) OLC with WT and mutant tau peptides at varying analyte concentrations. All peptides were immobilized at a fixed concentration (25 μM), and binding was analyzed using the titration cycle kinetics format, in which only analyte concentrations (PUR and OLC) were varied. Data were globally fitted to the kinetic titration model of global kinetic fitting to determine binding affinities (KD), association rate constant (Kon), and dissociation rate constant (Koff), and are summarized in Table 1. Source data details are provided in the Data Availability Statement section.

SPR was used to analyze the binding dynamics of OLC and PUR with monomeric WT and mutant tau peptides (Figure 2). The equilibrium dissociation constant (KD), association rate constant (Kon), and dissociation rate constant (Koff) were determined to characterize binding strength and stability.

PUR demonstrated moderate binding affinities to WT and mutant peptides (Table 1). For the WT peptide, the interaction was characterized by a KD of 4.35 × 10^−6^ M, a Kon of 60.95 M^−1^ s^−1^, and a Koff of 2.65 × 10^−4^ s^−1^, suggesting a moderately stable complex. Among the mutants, PUR exhibited stronger binding to N279K and V287I peptides (KD: 4.22 × 10^−6^ and 4.73 × 10^−6^ M, respectively) than to the P301L peptide (KD: 1.48 × 10^−5^ M). The reduced binding stability to the P301L peptide was evident from its higher dissociation rate (Koff: 3.90 × 10^−4^ s^−1^), suggesting that structural differences in this variant may reduce the stability of PUR binding, potentially diminishing its inhibitory efficacy.

**TABLE 1:** Binding affinities (KD), association rate constants (Kon), and dissociation rate constants (Koff) for purpurin (PUR) and oleocanthal (OLC) binding to R2R3 (wild‐type [WT] and mutant) peptides, determined from titration cycle kinetics sensorgrams. Lower KD values indicate stronger binding affinity.

Ligand	R2R3	KD (M)	Kon (M^−1^ s^−1^)	Koff (s^−1^)
PUR	WT	4.35 × 10^−6^	60.95	2.65 × 10^−4^
	P301L	1.48 × 10^−5^	26.37	3.90 × 10^−4^
	V287I	4.73 × 10^−6^	42.10	1.99 × 10^−4^
	N279K	4.22 × 10^−6^	70.34	2.97 × 10^−4^
OLC	WT	2.87 × 10^−6^	34.64	9.97 × 10^−4^
	P301L	1.58 × 10^−6^	52.36	8.26 × 10^−5^
	V287I	2.06 × 10^−6^	42.24	8.69 × 10^−5^
	N279K	1.98 × 10^−6^	34.60	6.87 × 10^−5^

OLC exhibited weaker binding affinity and less stable interactions with the WT peptide (KD = 2.87 × 10^−6^ M) relative to the mutants (Table 1). Its interaction with the WT peptide was characterized by a slower association rate (Kon: 34.64 M^−1^ s^−1^) and faster dissociation rate (Koff: 9.97 × 10^−4^ s^−1^), reflecting a less stable interaction. In contrast, mutant peptides exhibited moderately slower association rates (Kon: 52.36 M^−1^ s^−1^ for R2R3 [P301L], 42.24 M^−1^ s^−1^ for R2R3 [V287I], and 34.60 M^−1^ s^−1^ for R2R3 [N279K]) but markedly slower dissociation rates (Koff: 8.26 × 10^−5^ s^−1^ for R2R3 [P301L], 8.69 × 10^−5^ s^−1^ for R2R3 [V287I], and 6.87 × 10^−5^ s^−1^ for R2R3 [N279K]), indicating a more stable inhibitor‐peptide complex.

### 2.3 PUR and OLC preferentially inhibit fibrillization and aggregation kinetics of mutant tau peptides

**FIGURE 3:**
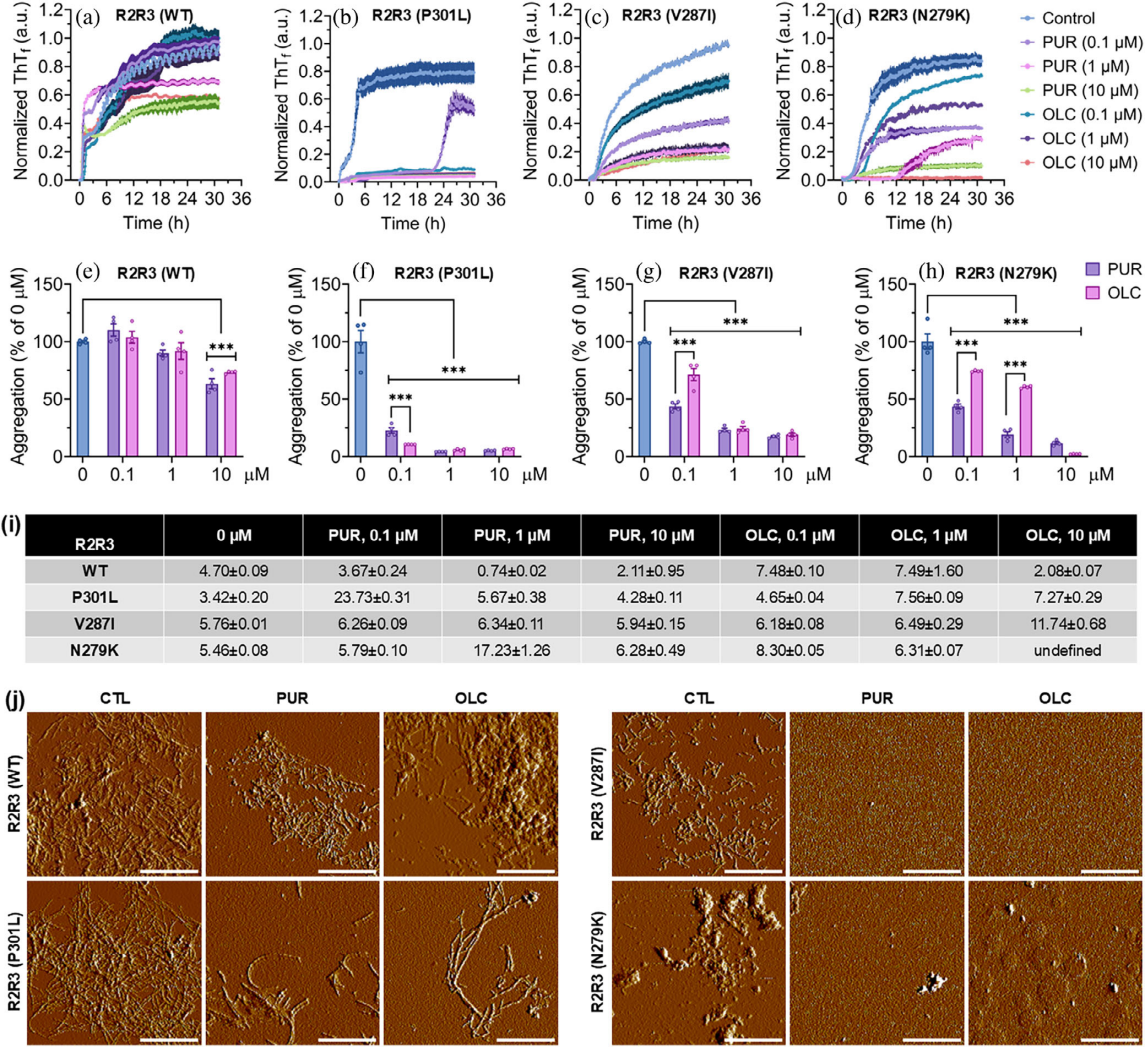
Purpurin (PUR) and oleocanthal (OLC) inhibit mutant tau peptide aggregation. (a–d) Thioflavin T (ThT) fluorescence kinetics of monomeric R2R3 peptides (wild‐type [WT] and mutants) incubated without (0 μM, control) or with increasing concentrations of PUR or OLC. Mean ± SEM (*n* = 3). Curves are normalized to the maximum signal of the untreated control (0 μM). Source data are provided in the Data Availability Statement section. (e–h) Effect of PUR and OLC on peptide aggregation, quantified by calculating the area under the curve from the ThT fluorescence curves. Mean ± SEM (*n* = 3). ***p < 0.001, two‐way ANOVA (Tukey's post hoc test). (i) Aggregation *t*_1/2_ derived from sigmoidal fits of individual replicates (*n* = 4). Mean ± SEM. Values are reported only for conditions where sigmoidal fitting was robust. No reliable fitting was obtained for R2R3 (N279K) treated with 10 μM OLC; the corresponding *t*_1/2_ is reported as “undefined.” Individual replicate curves with corresponding fitting functions are shown in Figures S5–S8. (j) Atomic force microscopy analysis of aggregates formed in the presence of 10 μM PUR or OLC. Control images for each peptide are reused from Figure 1d for direct morphological comparison. Scale bar: 1 μm.

Given the stronger binding affinities and more stable interactions of PUR and OLC with mutant tau peptides observed in the SPR analysis (Figure 2), we next investigated whether these interactions translated into enhanced inhibition of mutant tau aggregation. To evaluate this, monomeric R2R3 peptides were incubated with PUR or OLC at 0.1, 1, and 10 μM, corresponding to peptide:compound molar ratios of 500:1, 50:1, and 5:1, respectively (Figure 3a–d). No ThT fluorescence signal was detected without peptide, confirming that neither compound produced background fluorescence (Figure S4). Compound effects were evaluated by quantifying the area under the curve (AUC) of ThT fluorescence aggregation kinetics and determining the aggregation *t*_1/2_ from sigmoidal fits (Figures S5–S8).

Both compounds significantly reduced the aggregation of WT peptide at 10 μM compared to untreated controls, with no significant difference between them (63% for PUR vs. 73% for OLC; Figure 3e). However, both compounds exhibited substantially stronger inhibitory effects on mutant peptide aggregation at all tested concentrations, with apparent dose‐dependent inhibition observed for V287I and N279K peptides (Figure 3g,h).

For P301L peptide, PUR and OLC significantly suppressed aggregation across all concentrations, reducing aggregation levels below 10% at 10 μM (Figure 3f). At 0.1 μM, OLC exhibited greater suppression (8%) of aggregation than PUR (47%). However, PUR markedly delayed aggregation kinetics, showing a substantial increase in *t*_1/2_ (23.73 h) compared to OLC (4.65 h) relative to control (3.34 h) (Figure 3i). Induced fit docking (IFD) and molecular mechanics/generalized born surface area (MM‐GBSA) binding free energy calculations using AlphaFold2‐predicted tau peptide monomer structures revealed more favorable binding of both PUR and OLC to the P301L variant compared to WT (Tables S1 and S2 and Figure S10). Detailed docking scores and energy components (Table S1), MM‐GBSA binding free energies (Table S2), structural confidence assessments of the modeled tau peptides (Figure S9), and representative ligand‐residue interaction maps (Figure S10) are provided in Supporting Information S1.

Both compounds inhibited the aggregation of the V287I peptide across all concentrations (Figure 3g,i). At 0.1 μM, PUR was more effective than OLC (44% vs. 71% aggregation), although *t*_1/2_ values were similar (6.26 h for PUR and 6.18 h for OLC; Control: 5.76 h). Similarly, PUR demonstrated more potent inhibition of aggregation in N279K peptide at both 0.1 μM (44% vs. 74%) and 1 μM (19% vs. 61%) compared to OLC (Figure 3h). This trend was also evident in kinetics data, with a notably prolonged *t*_1/2_ at 1 μM PUR (19.87 h) compared to OLC (6.11 h; Figure 3i). Notably, no t_1/2_ was determined for OLC at 10 μM due to complete aggregation suppression, preventing sigmoidal fitting (Figure S8).

AFM imaging corroborated these findings, showing reduced fibril abundance after treatment with 10 μM PUR or OLC (Figure 3j). WT samples displayed shorter, fragmented fibrils, whereas P301L peptide samples showed only sparse fibrils. Following compound treatment, no fibrillar or aggregated structures were detected for V287I or N279K peptides. Similarly, fluorescence microscopy imaging of ThT‐stained aggregates from the reaction wells confirmed inhibition of fibril formation by PUR and OLC (Figure S11).

### 2.4 PUR and OLC inhibit the elongation of preformed WT and mutant tau peptide fibrils

**FIGURE 4:**
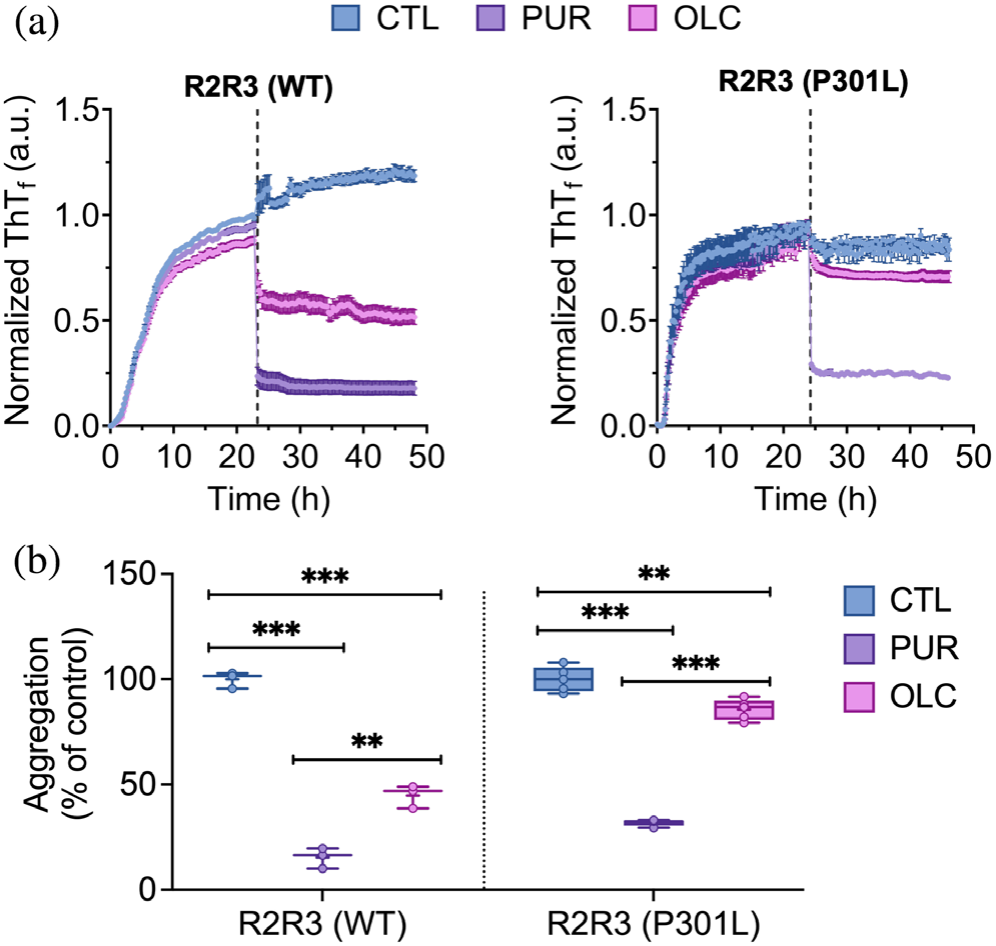
Purpurin (PUR) and oleocanthal (OLC) inhibit elongation of preformed wild‐type (WT) and P301L tau fibrils. (a) The thioflavin T (ThT) fluorescence kinetics of pre‐aggregated R2R3 (WT and P301L) tau peptides treated with PUR or OLC (10 μM). Peptides were first allowed to aggregate for 24 h, followed by compound addition and continued incubation for an additional 24–30 h. Vertical dashed lines indicate the time point of compound addition. Curves are normalized to each replicate's maximum pre‐treatment ThT fluorescence. Source data are provided in the Data Availability Statement section. Mean ± SEM (*n* ≥ 3). (b) Aggregation levels post‐treatment, expressed as a percentage of control (untreated fibrils), calculated from the area under the curve after compound addition. Mean ± SEM (*n* ≥ 3). ***p < 0.001, **p < 0.01, one‐way ANOVA (Tukey's post hoc test).

Given the favorable interactions with the monomeric form of P301L tau peptides, we next examined whether PUR and OLC could inhibit the elongation of preformed R2R3 fibrils (WT and P301L). Peptides were pre‐aggregated for 24 h with aggregation kinetics monitored by ThT fluorescence, after which 10 μM PUR or OLC was added, followed by an additional 24–30 h of incubation (Figure 4a). Aggregation kinetics was monitored by ThT fluorescence, and elongation was quantified as the AUC following compound addition, normalized to each replicate's maximum pre‐treatment fluorescence (Figure 4b). Both compounds suppressed fibril elongation, with PUR consistently more effective. WT peptide fibril elongation was reduced to 15.5 ± 2.8% (PUR) and 45.2 ± 3.2% (OLC), and P301L peptide fibril elongation was limited to 31.8 ± 0.6% (PUR) and 85.6 ± 2.2% (OLC) (Figure 4b). Fluorescence microscopy imaging further confirmed the presence of reduced ThT‐positive aggregates following the addition of PUR and OLC (Figure S12). Similar inhibition trends, with PUR outperforming OLC, were also observed for V287I and N279K peptides (Figure S12).

### 2.5 Molecular docking and simulation reveal preferential binding of PUR and OLC to mutant tau

**FIGURE 5:**
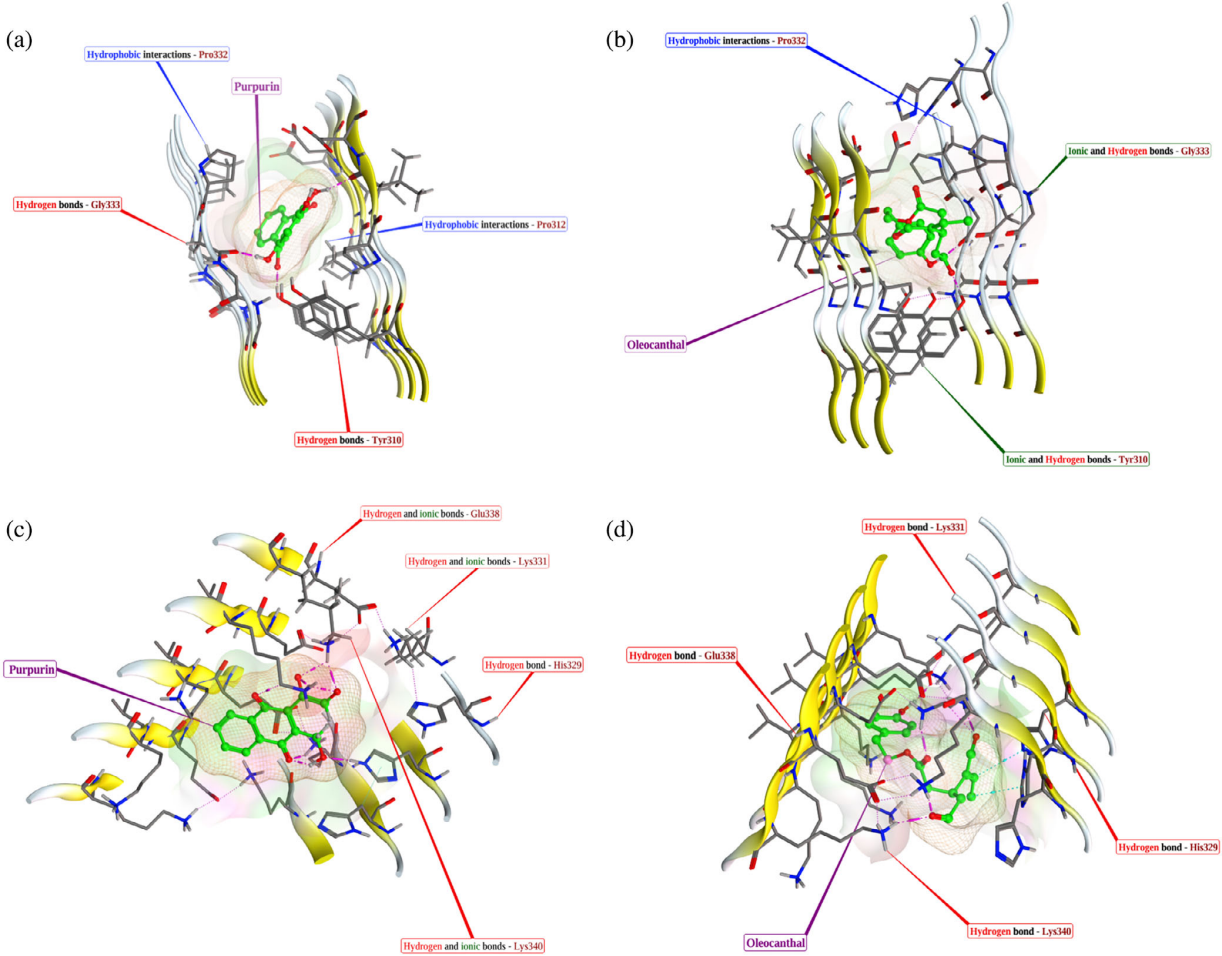
Docking interactions of purpurin (PUR) and oleocanthal (OLC) with wild‐type (WT) and P301L tau filament models. Predicted binding poses of PUR (a, c) and OLC (b, d) with the WT tau filament (PDB ID: 5O3L; panels a, b) and the P301L tau filament (PDB ID: 9GG0; panels c, d). Docking was performed using Schrödinger Release 2025‐1, and interactions were evaluated for hydrogen bonding, hydrophobic contacts, and ionic interactions. For corresponding molecular dynamics simulations of the 5O3L‐compound and 9GG0‐compound complexes, see Figures S14 and S15, respectively.

Molecular docking was performed for both compounds with WT (PDB ID: 5O3L) and P301L (PDB ID: 9GG0) tau filament structures obtained from the research collaboratory for structural bioinformatics (RCSB) Protein Data Bank. Docking scores indicated stronger predicted binding of both compounds to the P301L mutant (Table 2). Structural modeling showed that PUR binds to the WT tau filament via ionic and hydrogen bonds with LYS331, GLU338, and LYS340, and an additional hydrogen bond with HIS329 (Figure 5a), while OLC interacts with HIS329, LYS331, GLU338, and LYS340 through hydrogen bonds (Figure 5b). In the P301L filament, PUR forms hydrogen bonds with TYR310 and GLY333, and forms hydrophobic contacts with PRO312 and PRO332 (Figure 5c), while OLC engages in hydrogen bonding and ionic interactions with GLY333 and TYR310, along with hydrophobic contacts with PRO332 and TYR310 (Figure 5d).

**TABLE 2:** Induced fit docking (IFD) scores, Glide docking scores, and Prime energy values for purpurin (PUR) and oleocanthal (OLC) bound to tau filaments 5O3L (wild‐type tau) and 9GG0 (P301L mutant tau). Lower values indicate stronger binding affinity. Source data details are provided in the Data Availability Statement section.

Structure	Ligand	Glide score (kcal/mol)	Prime energy	IFD score
5O3L	OLC	−4.51	−27471.31	−1378.08
	PUR	−5.14	−27390.97	−1374.68
9GG0	OLC	−7.86	−12481.53	−631.93
	PUR	−8.43	−12417.20	−629.29

These docked complexes were used as starting structures for 100 ns molecular dynamics (MD) simulations to assess binding stability and dynamic interaction profiles. This timescale is generally sufficient to capture secondary structure formation, internal motions, and relevant domain fluctuations (Cino et al., 2012; Ekman et al., 2021). Protein–ligand interaction graphs from the simulations confirmed persistent hydrogen bonding, hydrophobic, and ionic contact across both systems (Figures S14 and S15). Subsequent MM/GBSA analysis performed on the equilibrated MD frames revealed markedly lower binding free energy (Δ*G* Bind) values for both compounds in complex with P301L tau (−50.15 to −50.60 kcal/mol) compared to WT tau (−21.99 to −24.61 kcal/mol) (Table 3). Binding free energy decomposition highlighted key electrostatic (Coulombic and hydrogen bonding) and lipophilic contributions in both WT and P301L tau complexes. Full datasets for MM/GBSA calculations and residue‐level interaction profiles are available in Supporting Information Datasets S1 and S2 and are openly accessible via Zenodo (see Data Availability Statement section).

**TABLE 3:** Binding free energy (Δ*G* Bind) and its contributing components were calculated using the molecular mechanics/generalized born surface area method. All energy values are in kcal/mol. Δ*G* Bind represents the total binding free energy, while Coulomb, covalent, H‐bond, lipophilic, solvation GB, and van der Waals indicate the individual energy contributions. Source data details are provided in the Data Availability Statement section.

Structure	Ligand	Δ*G* bind	Coulomb	Covalent	H‐bond	Lipophilic	Solvation GB	van der Waals
5O3L	OLC	−24.61	−10.66	0.17	−0.72	−6.89	13.19	−19.21
	PUR	−21.99	−6.01	0.36	−0.61	−3.63	15.84	−27.92
9GG0	OLC	−50.60	−22.75	1.52	−1.18	−13.91	18.89	−32.41
	PUR	−50.16	−7.90	0.86	−0.80	−14.91	12.04	−39.00

Abbreviations: OLC, oleocanthal; PUR, purpurin.

### 2.6 PUR and OLC reduce the formation of seeding‐competent aggregates

**FIGURE 6:**
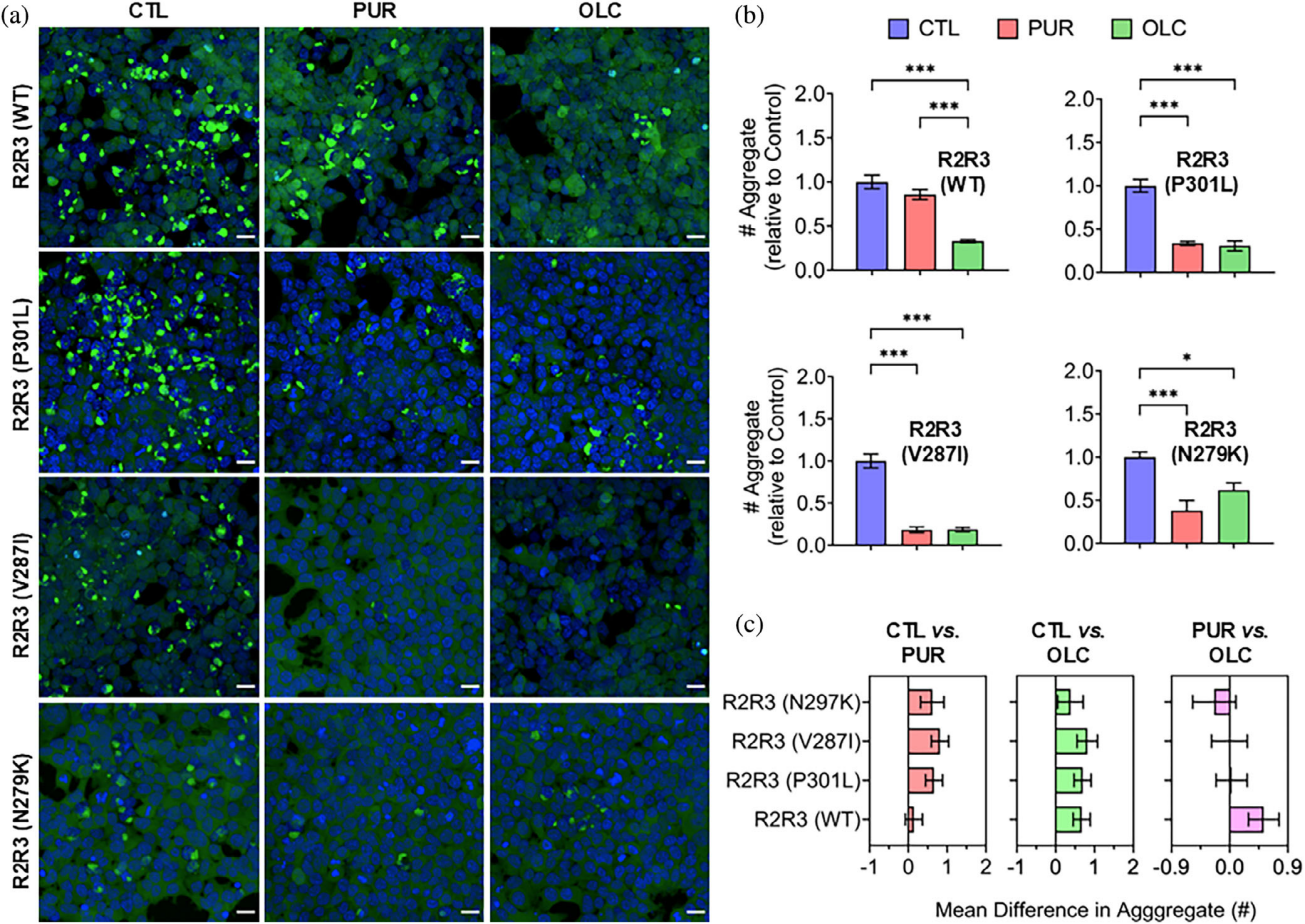
Oleocanthal (OLC) inhibits prion‐like aggregates of wild‐type (WT) and mutant tau peptides. (a) Confocal images of Tau RD P301S FRET Biosensor cells transduced with WT and mutant tau (R2R3) peptide aggregation products formed with or without purpurin (PUR) or OLC. Seeded Tau RD P301S in cells appear as cyan fluorescent protein/yellow fluorescent protein (CFP/YFP) (green) inclusions. Hoechst‐33342 (blue). Scale bar: 100 μm. (b) Quantification of CFP/YFP inclusions normalized to cell confluency. Mean ± SEM (*n* ≥ 3). ***p < 0.001, *p < 0.05, one‐way ANOVA (Tukey's post hoc test). Source data details are provided in the Data Availability Statement section. (c) Mean difference analysis showing the efficacy of PUR and OLC in preventing the conversion of WT and mutant R2R3 tau peptides into prion‐like forms compared to respective controls (left, middle) or relative to each other (right). Mean differences were derived from Tukey's multiple comparisons test (panel b). Error bars indicate 95% confidence intervals (CI), with non‐overlapping 95% CIs indicating statistically significant differences.

To evaluate the biological relevance of the anti‐aggregation effects observed in vitro, we transduced Tau RD P301S FRET Biosensor cells with aggregation reaction products from control and compound‐treated WT and mutant peptides. Intracellular seeding was determined by quantifying the seeded cyan fluorescent protein/yellow fluorescent protein (CFP/YFP) inclusions of Tau RD P301S by confocal microscopy (Annadurai, Malina, Salmona, et al., 2022). OLC significantly reduced the seeding competency of WT peptide aggregates, effectively abolishing intracellular seeding (Figure 6a,b). In contrast, PUR did not affect the seeding competency of WT peptide aggregates. However, for mutant tau peptides, both compounds abolished the formation of species capable of inducing intracellular seeding (Figure 6a,b).

For R2R3 (P301L), PUR and OLC significantly reduced seeding, with comparable efficacy between the two compounds (Figure 6c). For V287I peptide aggregates, both compounds exhibited near‐complete inhibition of seeding, with mean difference analysis showing negligible differences between their effects. Similarly, for N279K peptide aggregates, both compounds significantly reduced seeding. However, PUR showed slightly more significant inhibition than OLC (Figure 6c).

### 2.7 PUR and OLC inhibit tau seeding when administered after aggregate transduction

**FIGURE 7:**
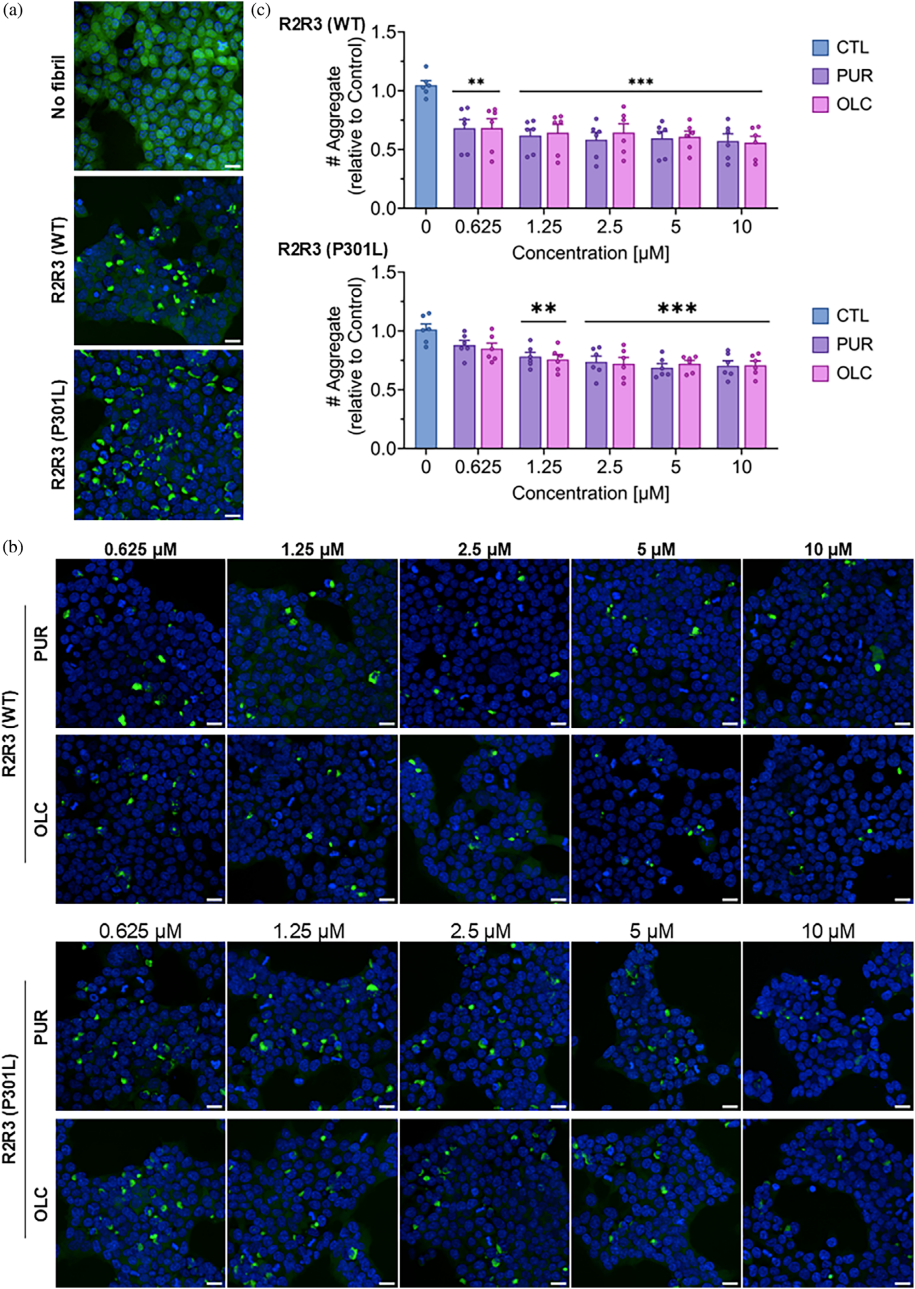
Purpurin (PUR) and oleocanthal (OLC) inhibit pathological tau propagation in cells pre‐treated with wild‐type (WT) and P301L peptide aggregates. (a) Representative images of Tau RD P301S FRET Biosensor cells showing no intracellular aggregates in untransduced cells (no fibril), while cells transduced with R2R3 (WT) or R2R3 (P301L) fibrils display abundant seeded cyan fluorescent protein/yellow fluorescent protein (CFP/YFP) (green) inclusions after 48 h (*n* = 3). (b) Treatment of fibril‐transduced cells with PUR or OLC at the indicated concentrations for 24 h markedly reduces the number of seeded inclusions. (c) Quantification of seeded aggregates (CFP/YFP inclusions) in cells transduced with R2R3 (WT) or R2R3 (P301L) fibrils for 24 h, followed by compound treatment for 24 h. Mean ± SEM (*n* = 3). ***p < 0.001, **p < 0.01 versus 0 μM (CTL), two‐way ANOVA (Šídák's post hoc test). Nuclei are stained with Hoechst‐33342 (blue). Scale bar: 20 μm (a, b). Source data are provided in the Data Availability Statement section.

To further evaluate whether PUR and OLC can suppress pathological tau propagation, we performed post‐transduction treatment assays in Tau RD P301S FRET Biosensor cells. WT or P301L tau fibrils were first transduced into cells for 24 h, followed by treatment with PUR or OLC for an additional 24 h. Prior MTS assays confirmed that these concentrations were non‐toxic to biosensor cells (Figure S16). Compared to fibril‐only, drug‐untreated controls (Figure 7a), drug‐treated cells exhibited a marked reduction in intracellular CFP/YFP inclusions (Figure 7b). Quantification confirmed that both PUR and OLC significantly reduced seeding at all tested concentrations for WT fibrils and at concentrations ≥1.25 μM for P301L fibrils (Figure 7c). Two‐way analysis of variance (ANOVA) revealed a significant effect of concentration (*p* < 0.001) but no significant difference between PUR and OLC for either WT or P301L. Neither compound induced endogenous tau aggregation when applied alone, as no FRET‐positive inclusions were detected without exogenous seeds (Figure S17).

### 2.8 OLC reduces the pathogenicity of P301L peptide aggregates in SY5Y‐TauP301L‐EGFP cells

**FIGURE 8:**
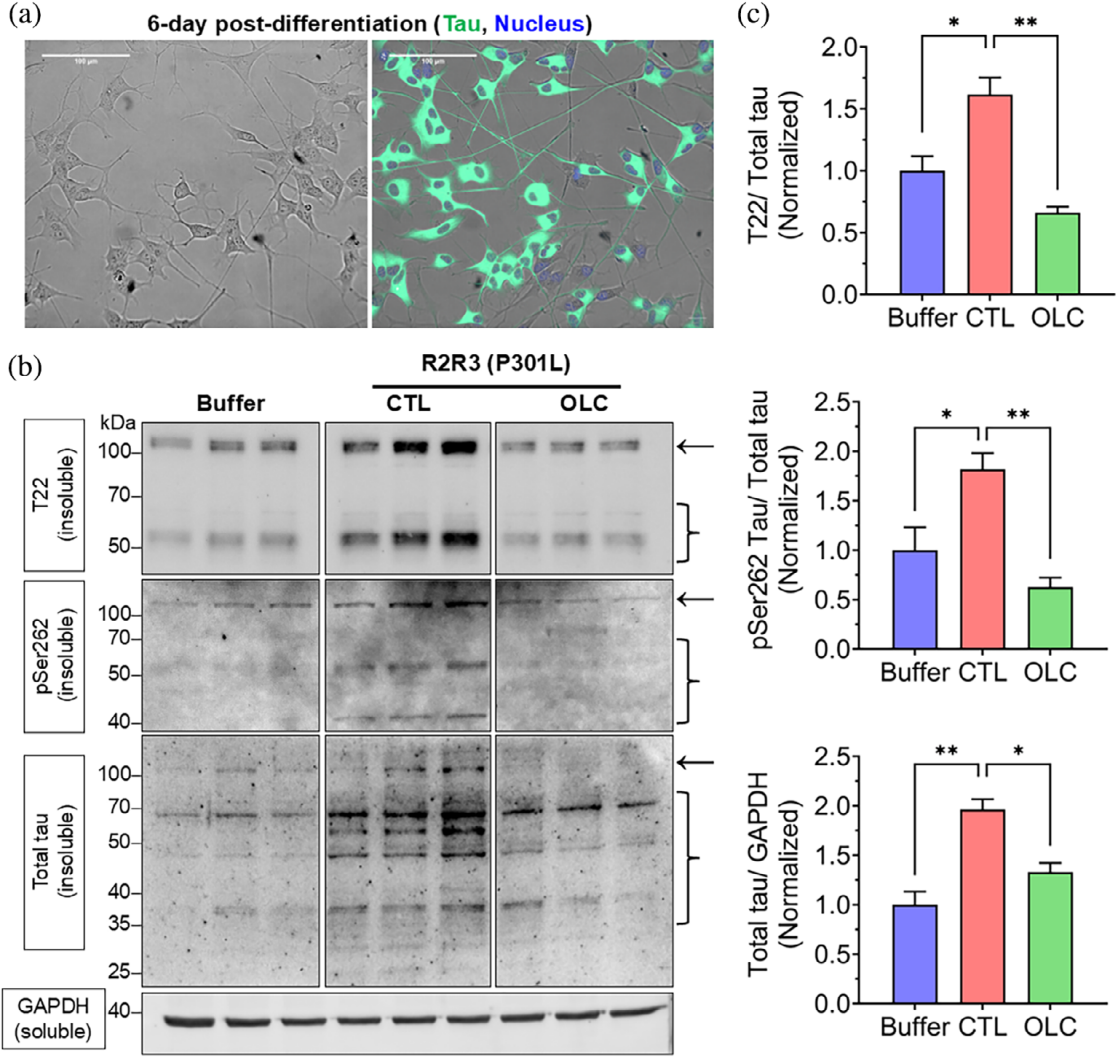
Oleocanthal (OLC) prevents P301L tau peptide aggregate‐induced phosphorylation and oligomerization. (a) Phase contrast and fluorescence microscopy images of differentiated SY5Y‐TauP301L‐EGFP cells showing the distribution of Tau (P301L) (green) following induction with doxycycline and nuclear staining (Hoechst, blue). Scale bar: 100 μm. (b) Western blot analysis of insoluble fractions (pSer262, T22, total tau) from cells transduced with R2R3 (P301L) tau peptide aggregated in the absence (control) or presence of OLC. GAPDH from soluble fractions was used as a loading control. Buffer: Cells transduced with the reaction mixture without the aggregation reaction product. Arrows indicate the position of TauP301L‐EGFP, expressed from the transfected plasmid, whereas brackets indicate endogenous wild‐type (WT) tau. Full‐length images of blots are shown in Figure S21. (c) Quantification of pSer262, T22, and total tau in the insoluble fraction. Signals were normalized to GAPDH; pSer262 and T22 were additionally normalized to total tau and shown as fold change vs. buffer control. Mean ± SEM (*n* = 3). **p < 0.01, *p < 0.05, one‐way ANOVA (Tukey's post hoc test).

Given OLC's broad inhibition of tau seeding across WT and mutant peptides in biosensor cells, and its known ability to covalently modify lysine residues near the amyloidogenic motifs (Monti et al., 2011; Monti et al., 2012), we further explored its impact on tau propagation. Specifically, we tested whether P301L peptide aggregates formed in its presence lose their ability to induce intracellular tau conversion into pathological forms. To assess this, differentiated SY5Y‐TauP301L‐EGFP cells were transduced with P301L peptide aggregation reaction products formed with or without OLC (Figure 8a).

To confirm that the high molecular weight (HMW) tau bands detected in the insoluble fractions originated from the transfected plasmid, whole cell lysates from SY5Y‐TauP301L‐EGFP cells were analyzed alongside those from parental SY5Y cells. Only SY5Y‐TauP301L‐EGFP cells exhibited HMW tau bands (Figure S18), aligning with the original study that reported the plasmid (Wang et al., 2020).

Next, we monitored changes in these HMW tau bands in fibril‐transduced SY5Y‐TauP301L‐EGFP cells. Cells transduced with P301L tau peptide aggregates showed a significant increase in insoluble total tau levels compared to buffer‐only transduced cells, confirming that exogenous fibrils effectively seeded intracellular tau aggregation (Figure 8b,c). This aggregation was accompanied by elevated tau phosphorylated at serine 262 (pSer262) and oligomeric tau T22 levels (Figure 8b,c). However, cells transduced with aggregation products formed with OLC exhibited significantly reduced pSer262, T22, and total tau levels (Figure 8b,c). Analysis of soluble fractions showed no significant differences in pSer262, T22, or total tau levels across treatments (Figure S19), supporting the compartmentalized nature of tau pathology, where pathological modifications predominantly occur in insoluble aggregates.

Additionally, WT peptide aggregates failed to induce significant tau pathology in SY5Y‐TauP301L‐EGFP cells. Analyses of soluble and insoluble fractions revealed no substantial differences in T22 or pSer262 tau between control and OLC‐treated samples, indicating the minimal impact of WT peptide aggregates on intracellular tau pathology (Figure S20).

## 3 DISCUSSION

This study demonstrates that PUR and OLC inhibit mutant tau aggregation and propagation, with OLC broadly reducing fibril formation across all tested tau mutants, while PUR is more selective for tau N279K and V287I over P301L peptides. Both compounds disrupt seeding‐competent tau aggregates, reducing intracellular pathology. These findings suggest distinct inhibitory profiles: OLC broadly suppresses tau aggregation by interacting with monomeric tau, while PUR more effectively inhibits fibril elongation through stable interactions with fibril surfaces.

The observed accelerated aggregation of P301L peptide aligns with previous studies using recombinant tau studies, confirming the strong fibrillizing tendency of this mutation (Yao et al., 2020). The slower aggregation kinetics of N279K peptides are consistent with reports that this mutation disrupts nucleation by altering hydrophobic interactions within the VQIIKK motif (Yao et al., 2003). In contrast, although V287I tau exhibited minimal aggregation in cellular models (Xia et al., 2019), our peptide model showed enhanced aggregation kinetics relative to WT. This discrepancy likely reflects differences between minimal peptide segments and full‐length tau in a cellular environment. Nonetheless, these peptides are relevant models for studying mutation‐specific tau fibrillization.

SPR studies supported prior reports that OLC interacts stably with tau (Li et al., 2009). Its stronger binding affinities for monomeric forms of P301L, V287I, and N279K peptides suggest that these mutations expose favorable interaction sites, enhancing OLC's inhibitory effects (Pounot et al., 2024). ThT aggregation assay confirmed that stronger tau–ligand interactions correlate with reduced fibril formation. At 10 μM (5:1 peptide:compound molar ratio), both compounds robustly inhibited aggregation below 10% for P301L peptide and strongly suppressed V287I and N279K peptides. In contrast, inhibition of WT aggregation was more modest, consistent with its lower SPR binding affinity.

Prior studies indicated that PUR inhibits PHF6 aggregation dose‐dependently (Viswanathan et al., 2020), with reduced efficacy at higher peptide:compound ratios such as 5:1. In contrast, we observed that PUR remained effective even at peptide:compound ratios up to 500:1, with measurable activity at concentrations as low as 0.1 μM. This broader activity range aligns with its SPR binding profile, which showed weaker affinity for P301L but stronger and more stable interactions with N279K and V287I peptides. In contrast, OLC demonstrated broad‐spectrum inhibitory effects across all mutant peptides, consistent with its slower Koff and lower KD values.

Although computational docking was limited to WT and P301L tau peptide monomers, the predicted binding affinities support the experimental observations. Both PUR and OLC showed stronger binding to P301L than to WT in docking and MM‐GBSA calculations, consistent with their enhanced inhibition of P301L aggregation and higher SPR affinity. These results suggest that the compounds may interfere with early nucleation steps by preferentially engaging aggregation‐prone conformations in mutant tau.

The ability of small molecules to inhibit tau aggregation also depends on their capacity to engage and disrupt growing or preformed fibrillar structures. PUR and OLC inhibited the elongation of preformed tau fibrils, with PUR consistently demonstrating stronger efficacy than OLC, particularly in WT and P301L peptides. These findings suggest that PUR more effectively engages fibril surfaces or occludes propagation‐competent sites, thereby impeding template‐directed elongation.

Computational analyses reinforce these experimental observations, indicating that lower binding free energy correlates with more potent inhibitory activity. Molecular docking and MM/GBSA simulations revealed that PUR and OLC interact favorably with fibrillar tau, with lower binding free energies for the P301L than WT. These non‐covalent interactions included hydrogen bonding, hydrophobic contacts, and ionic interactions. Notably, PUR formed a more extensive hydrogen bond network and stable hydrophobic contacts at surface‐exposed residues of the P301L filament, suggesting it may interfere with protofilament extension. In contrast, OLC formed fewer stabilizing contacts, and more transient ionic interactions dominated its binding.

These mechanistic insights align with the differential effects observed in aggregation and elongation assays: PUR's more potent inhibition of P301L elongation corresponds with its more favorable binding energies and stable interactions, whereas OLC's comparatively weaker inhibition is consistent with more transient binding interactions. Although computational analyses were limited to P301L tau, SPR data support PUR's mutation‐specific binding, with weaker affinity for P301L but stronger interactions with N279K and V287I tau peptides. These differences align with prior studies showing that structural variations among tau mutants alter their aggregation pathways and inhibitor activity (von Bergen et al., 2001).

Our experimental and computational approaches addressed tau interactions across multiple aggregation stages. SPR and ThT assays (with compound addition at time zero) modeled inhibition at the nucleation stage, while molecular docking and elongation assays examined compound effects on preformed fibrils. Together, these complementary strategies provide a comprehensive view of tau aggregation inhibition and fibril destabilization mechanisms. The selective inhibition observed for V287I and N279K may arise from local structural effects introduced by these mutations, which likely modulate the accessibility of aggregation‐prone motifs such as PHF6 and PHF6*. V287I, positioned near PHF6, may promote early β‐structure formation that facilitates PUR binding. In contrast, N279K lies within PHF6*, and its slower aggregation kinetics suggest an alternative pathway that may generate intermediates more susceptible to PUR. These differences in local sequence context and aggregation dynamics likely contribute to the distinct inhibitory profiles observed for PUR and OLC.

Unlike many tau inhibitors targeting generic fibrillization (Soeda & Takashima, 2020), OLC and PUR selectively inhibit seeding‐competent aggregates, a key driver of tau pathology. Small molecules such as methylene blue and TRx0237 have shown limited success in preventing tau propagation (Soeda et al., 2019; Soeda & Takashima, 2020). OLC's broad inhibition and PUR's selectivity suggest distinct therapeutic strategies may be needed for different tauopathies.

Our cell‐based studies confirmed that the inhibition of tau aggregation observed *in vitro* translated into reduced intracellular pathology. OLC showed broad efficacy, while PUR was particularly potent against V287I and N279K tau peptides. Notably, WT peptide aggregates failed to seed pathology in full‐length P301L tau‐expressing cells, aligning with prior reports that WT tau fibrils do not efficiently recruit mutant tau monomers due to structural incompatibility (Aoyagi et al., 2007). However, all tau peptide aggregates, including WT, seeded aggregation in Tau RD P301S FRET Biosensor cells, likely due to increased cross‐seeding permissibility in truncated tau constructs (Holmes et al., 2014), which has been used in multiple studies to detect seeding from tau species with diverse conformations and disease origins (DeVos et al., 2017; DeVos et al., 2018; Seidler et al., 2019). Importantly, both PUR and OLC significantly reduced intracellular seeding when administered 24 h after fibril transduction, demonstrating their ability to suppress tau propagation in the presence of pre‐existing aggregates. This treatment condition more accurately reflects the pathological context of tauopathy, where seed‐competent tau species have already accumulated, and supports the potential of these compounds to remain effective when administered after disease onset.

Despite strong mechanistic insights, this study has limitations. Computational analyses were restricted to WT and P301L tau, leaving molecular interactions for V287I and N279K tau mutants unmodeled. Additionally, only P301L, N279K, and V287I tau mutations were tested, and future work should evaluate efficacy across a broader range of tau mutations. Another key limitation is the lack of *in vivo* pharmacokinetic data to determine whether PUR and OLC cross the blood–brain barrier (BBB) at therapeutic concentrations. While *in vitro* studies suggest that PUR can permeate the BBB (Viswanathan et al., 2020), and OLC‐rich extra‐virgin olive oil has been shown to restore BBB function in mouse models (Al Rihani et al., 2019), direct evidence of their BBB permeability in humans is lacking. Future studies should assess BBB permeability, metabolic stability, and potential off‐target effects. Finally, validation in transgenic tauopathy models is needed to confirm their therapeutic potential in a biologically relevant system. Additionally, validation of aggregation inhibition in primary or iPSC‐derived neuronal models and investigation of downstream cellular effects such as neurotoxicity, proteasomal degradation, and autophagy modulation will further strengthen the translational relevance of these findings.

## 4 CONCLUSION

This study identifies PUR and OLC as mutation‐selective inhibitors of tau aggregation that suppress fibrillization, inhibit elongation, and reduce the formation of seeding‐competent aggregates. Both compounds showed preferential binding to mutant tau peptides, supported by SPR and computational modeling. Post‐transduction treatment assays demonstrated their ability to inhibit tau propagation even in the presence of pre‐existing aggregates, and additional biophysical validation confirmed their impact on fibril morphology. These findings support the potential of PUR and OLC as structurally distinct candidates for mutation‐specific therapeutic strategies in tauopathies.

## 5 MATERIALS AND METHODS

### 5.1 Materials

PUR (Part # PHL89771) and OLC (Part # SMB00810) were purchased from Sigma‐Aldrich (St. Louis, MO, USA), dissolved in high‐performance liquid chromatography (HPLC)‐grade dimethyl sulfoxide (DMSO), and stored at −80°C. Intermediate dilutions were prepared in phosphate‐buffered saline (PBS) (pH 7.4) supplemented with 0.05% Tween‐20 and 5% DMSO.

All peptides were custom‐synthesized at 98% purity by ProteoGenix (Schiltigheim, France) and verified by analytical HPLC and mass spectrometry by the supplier. Peptides were dissolved in double‐distilled water according to the supplier's recommendation. The stock solutions were prepared at a final concentration of 154 μM, immediately aliquoted into smaller volumes to prevent repeated freeze–thaw cycles, and stored at −80°C until use to minimize pre‐aggregation. Before each experiment, peptides were thawed on ice and briefly centrifuged at 13,000 rpm for 1 min. The monomeric state of peptides was confirmed by UV–Vis spectrometry and SDS‐PAGE analysis under non‐reducing conditions (Figures S1 and S2).

### 5.2 ThT aggregation assay

The aggregation kinetics of tau peptides were monitored using a ThT assay. A 40 μL reaction mixture containing 15 μM ThT (Sigma‐Aldrich, Cat. # T3516‐5G) and 50 μM peptide in the absence (control) or presence of 0.1 μM, 1 μM, and 10 μM PUR or OLC was prepared on ice in aggregation buffer (20 mM Tris [pH 7.4], 100 mM NaCl, 1 mM ethylenediaminetetraacetic acid [EDTA]) containing 5 μM heparin (Sigma‐Aldrich, Cat. #H4784). dithiothreitol (DTT) was excluded to avoid its differential effects on the aggregation kinetics of tau peptides containing Cys291 or Cys322 and to prevent peroxide generation by redox‐active compounds like PUR, which can lead to oxidative artifacts and false‐positive inhibition signals, as reported in our previous study (Annadurai, Malina, Salmona, et al., 2022). This mixture was then dispensed into a clear‐bottom, black 384‐well ViewPlate (Revvity, Waltham, MA, United States; Part # 6007460) and sealed with a TopSealA‐PLUS (Revvity) to prevent evaporation.

Aggregation was monitored using EnSpire Multimode Plate Reader (Revvity) at 37°C with constant agitation (1000 rpm). To minimize condensation, the upper heater temperature was set to 2°C higher than the lower heater temperature. ThT fluorescence (ThT—*λ*_ex_: 460–490 nm and *λ*e_m_: 500–550 nm) was recorded every 5–10 min for 48 h.

### 5.3 Aggregation *t*_1/2_ and AUC quantification

ThT fluorescence curves were fitted to a sigmoidal function in GraphPad Prism (version 10, San Diego, CA, USA) using the following equation:

$Y=\mathrm{Top}/\left(1+\exp\left[\left(V50–X\right)/\text{Slope}\right]\right)$, where *Y* is the ThT fluorescence intensity, *X* is time (h), Top is the maximum fluorescence intensity, V50 is the time to reach 50% of Top, and Slope describes the steepness of the aggregation transition. Baseline fluorescence at the start of the measurements was relatively low across samples and was not separately fitted. In samples showing early increases in fluorescence, the aggregation trajectory was modeled directly without introducing an independent baseline parameter.

The V50 value obtained from each replicate was used as the aggregation *t*_1/2_ for statistical analysis. In conditions where aggregation was completely inhibited and no sigmoidal trajectory was observed, *t*_1/2_ was conservatively assigned as >48 h, corresponding to the maximum assay duration.

To evaluate the effect of compounds on the extent of aggregation, the AUC was calculated for each replicate ThT fluorescence curve using GraphPad Prism. AUC values were then normalized to the mean AUC of the untreated control group and expressed as “aggregation (% of control),” serving as an indirect and comparative measure of fibril formation.

### 5.4 Atomic force microscopy

Aggregates collected after 48 h of aggregation were processed for AFM analysis as described previously (Annadurai, Malina, Malohlava, et al., 2022).

### 5.5 SPR experimental setup and data collection

SPR experiments were conducted using the Sierra® SPR‐24 Pro system (Bruker Daltonics, Bremen, Germany), featuring a gold‐coated microfluidic array (24 detection spots, 3 × 8 format). Data acquisition and kinetic analysis were performed using SPR Analyzer 4 software.

All experiments were conducted at 25°C using PBS (pH 7.4) supplemented with 0.05% Tween‐20 and 5% DMSO as the running buffer to ensure assay reproducibility. Assay components, including 96‐well microtiter plates (Part # 1862984; Burker) and Microplate and Reservoir Sealer (Part #1862985; Burker), were used for sample handling and were compatible with the Sierra SPR®‐24 Pro system (Bruker Daltonics, Billerica, MA, USA).

### 5.6 Sensor surface preparation and immobilization

WT and mutant (P301L, V287I, and N279K) tau peptides were dissolved in 10 mM sodium acetate buffer (pH 5.0; Burker) and immobilized onto High‐Capacity Amine Sensor Surfaces (Part #1862614; Bruker) using the Amine Coupling Kit (Part #1862634; Bruker). The sensor surface was preconditioned with a Surface Elution Buffer (Part #1862656; Bruker Daltonics) and 0.1M HCl to remove contaminants and improve binding specificity.

The surface was activated using 400 mM 1‐ethyl‐3‐(3‐dimethylaminopropyl) carbodiimide (Burker) and 50 mM *N*‐hydroxysuccinimide (Burker), enabling covalent attachment of tau peptides. Unreacted binding sites were blocked with 1M ethanolamine‐HCl (pH 8.5; Burker) to minimize nonspecific interactions. For each experiment, specific sensor spots (A/B/C) on channels 1–8 were designated for tau‐analyte interactions, with corresponding reference spots on the same channel for baseline correction. DMSO calibration solutions (4.6%, 4.8%, 5%, 5.2%, and 5.4%) were prepared for double referencing to compensate for DMSO solvent effects.

### 5.7 Analyte preparation and injection

PUR and OLC were prepared in PBS (pH 7.4) supplemented with 0.05% Tween‐20 and 5% DMSO. Compound interactions with tau peptides were pre‐screened using a concentration range of 1–100 μM, and subsequent binding kinetics were evaluated at optimized concentrations. For kinetic studies, PUR was tested at 20–30 μM, while OLC was tested at 25–100 μM using two‐fold serial dilutions. Each analyte was sequentially injected, and association and dissociation phases were monitored to determine kinetic parameters.

### 5.8 Titration cycle kinetics assay

SPR binding kinetics were assessed using the titration cycle kinetics (TCK) assay, which included consecutive association phases followed by a single dissociation phase. Pre‐assay simulations using the Kinetic Simulator feature of SPR Analyzer 4 software were used to optimize analyte concentration ranges and association/dissociation times. A reference blank injection was included for double referencing, and a single dissociation curve was recorded for the highest analyte concentration.

### 5.9 SPR data analysis and validation

Sensorgrams were analyzed using the Kinetic Titration model, which was integrated into the global kinetic fitting model of the SPR Analyzer 4 software. This approach allowed for baseline correction and improved the reliability of kinetic parameters, including the Kon, Koff, and KD.

TCK‐derived kinetic parameters were compared across multiple runs and confirmed using Multi Injection Cycle Kinetics to validate reproducibility and accuracy. The TCK format provided comparable results with reduced assay time and lower reagent consumption.

### 5.10 Computational methods

#### 5.10.1 Protein and ligand preparation

The atomic structures of tau filaments were obtained from the RCSB protein data bank (https://www.rcsb.org/), specifically 5O3L (paired helical filaments from AD brain) and 9GG0 (P301L tau filaments from FTD brain). The protein structures were prepared using the Protein Preparation Wizard in Schrödinger Maestro 2025‐1 (Schrödinger, 2025e). Pre‐processing steps included adding hydrogen atoms, completing missing side chains and loops via Prime refinement, optimizing hydrogen bonding networks with PROPKA at pH 7.4, and minimizing energy using the OPLS4 force field. Water molecules beyond 5 Å from the binding site were removed to improve docking accuracy.

PUR (PubChem CID: 6683) and OLC (PubChem CID: 11652416) were prepared using LigPrep in Schrödinger Maestro 2025‐1 (Schrödinger, 2025a). The ligands were optimized at pH 7.4, generating the lowest‐energy stereoisomers and tautomers. Energy minimization was performed using the OPLS4 force field to obtain stable conformations for molecular docking.

#### 5.10.2 Molecular docking

Molecular docking was performed using IFD in Schrödinger Maestro 2025‐1 to predict the binding interactions of PUR and OLC with tau filaments (Schrödinger, 2025b). Ligands were initially docked into the receptor binding site using Glide Standard Precision (SP) mode, followed by side‐chain refinement with Prime to optimize receptor flexibility (Schrödinger, 2025c). The optimized ligand poses were redocked using Glide Extra Precision (XP), and docking scores were generated based on interaction energies (Friesner et al., 2006). Protein–ligand interactions, including hydrogen bonding, hydrophobic interactions, and salt bridges, were analyzed to determine binding affinity and specificity.

#### 5.10.3 Molecular dynamics simulations

MD simulations were conducted using Desmond in Schrödinger Maestro 2025‐1 to assess the stability of the docked ligand–protein complexes (Schrödinger, 2025d). The systems were solvated in an SP3 water box, neutralized with Na^+^ and Cl^−^ ions, and equilibrated under periodic boundary conditions. The simulations were run for 100 ns at 300 K and 1 atm under contant number of particles, pressure, and temperature using the OPLS4 force field. The stability of ligand interactions was analyzed using Simulation Interaction Diagram (SID) analysis, classifying interactions into hydrogen bonds, hydrophobic contacts, ionic bonds, and water bridges.

#### 5.10.4 Binding free energy calculations

Binding free energy (Δ*G*_bind) was calculated using the MM/GBSA method in Schrödinger Maestro 2025‐1 (Genheden & Ryde, 2015). Energy contributions from van der Waals interactions, electrostatics, solvation effects, and entropy were considered. The MM/GBSA‐derived binding energy values were used to estimate the relative affinity of PUR and OLC for tau filaments.

### 5.11 Cell culture

Tau RD P301S FRET Biosensor (expressing tau repeat domain fused to CFP/YFP for detecting intracellular tau aggregation) and SH‐SY5Y cells were purchased from ATCC (Manassas, VA, USA). Both cell lines were cultured in 89% Dulbecco's Modified Eagle's Medium (DMEM) high (4.5 g/L) glucose with L‐Glutamine (Lonza, # 12‐604F), supplemented with 10% fetal bovine serum (Gibco, Cat. # A5256701), and 1% Penicillin–Streptomycin (Thermo Fisher Scientific, Cat #15140163). HEK293T biosensor cells were further supplemented with 1 mM GlutaMAX™ (Thermo Fisher Scientific Inc., Cat. # 35050061) and 10 mM 4‐(2‐hydroxyethyl)‐1‐piperazineethanesulfonic acid (HEPES) (Serana Europe GmbH, Brandenburg, Germany; Cat. # BSL‐001‐100ML). All cells were maintained in a 5% CO_2_ humidified incubator at 37°C and split every 3–4 days after reaching ~80% confluency. Cells were routinely authenticated and tested for mycoplasma contamination.

### 5.12 Preparation of samples for cell seeding assays

Tau peptides were aggregated with or without PUR or OLC in a 7.4 pH aggregation buffer without ThT. After 48 h, the contents of each well were pooled to a final total volume of 120 μL. The concentration of the pooled Aggregation Reaction Products was measured using the Pierce™ BCA Protein Assay Kit (Thermo Fisher Scientific Inc., Cat. # 23225). The reaction products were standardized for cell transduction to a final concentration of 100 nM for both control and drug‐treated samples. The pooled aggregation mixture was sonicated using a Branson Ultrasonic™ Sonifier Cup Horns (Marshall Scientific, Hampton, NH, USA) at 30% amplitude (15 s ON and 15 s OFF for 1 min) to fragment larger aggregates into smaller ones, facilitating consistent transduction.

### 5.13 Biosensor cell seeding assay

Tau RD P301S FRET Biosensor cells were plated at 5 × 10^3^ cells per well in PhenoPlate 384‐well, black, optically clear flat‐bottom plates (Revvity; Part # 6057302) pre‐coated with 50 μg/mL Poly‐ᴅ‐Lysine (Sigma‐Aldrich, Cat. # P6407). After plating, cells were incubated for 30 min at room temperature to promote better cell spreading before returning the plates to the incubator.

The next day, cells were transduced with 100 nM of aggregation mixture, which were either: (i) prepared in the absence (control) or presence of 10 μM PUR or OLC for co‐aggregation studies (Figure 5), or (ii) prepared exclusively in the absence of compounds for post‐transduction treatment studies (Figure 6). The aggregation mixtures were premixed with 0.2 μL per well TurboFect™ Transfection Reagent (Thermo Fisher Scientific Inc., Cat. # R0533) in 10 μL Opti‐MEM™ (Thermo Fisher Scientific Inc., Cat. #11058021) and added to cells.

For co‐aggregation studies (Figure 5), cells were cultured for 48 h after transduction before processing for imaging. For post‐transduction treatment studies (Figure 6), cells were cultured for 24 h following transduction, then treated with PUR or OLC at concentrations ranging from 0 to 10 μM, and incubated for an additional 24 h.

In both experiments (Figures 5 and 6), cells were fixed after a total of 48 h with 2% paraformaldehyde (Electron Microscopy Services, Cat. #15714‐1 L) for 10 min at room temperature, washed once with 1× PBS, and stained with 10 μM Hoechst‐33342 nuclear dye (Invitrogen, Cat. #H21492) for 10–15 min. To preserve samples for imaging, 1% glycerol in deionized water was added to each well.

### 5.14 Imaging and quantification

Cells were imaged on a Cell Voyager 7000S high‐content imaging system (Yokogawa, Tokyo, Japan) with a 20× objective. CFP/YFP inclusions of seeded tau were detected using a 488 nm laser line (Ex = 460–490 nm, Em = 500–550 nm), and Hoechst‐stained nuclei were detected using a 405 nm laser line (Ex = 360–400 nm, Em = 410–480 nm). At least 10–15 focal areas per well were imaged for each replicate. Image analysis was performed using Signals Image Artist (Revvity) to quantify the number of cells (Hoechst‐stained nuclei) and intracellular CFP/YFP inclusions. Total inclusions per well were normalized to cell confluence and are presented as normalized seeding for graphing.

### 5.15 SH‐SY5Y plasmid transfection and differentiation

SH‐SY5Y cells were transfected with the AAVS1 CAG rtTA3 TauP301L 2N4R‐EGFP plasmid using ScreenFect‐A‐plus Transfection Reagent (ScreenFect GmbH). AAVS1 CAG rtTA3 TauP301L 2N4R‐EGFP was a gift from Gerold Schmitt‐Ulms (Addgene plasmid #132393; http://n2t.net/addgene:132393; RRID: Addgene_132393). Stable cell lines expressing TauP301L 2N4R‐EGFP were generated via puromycin selection and are referred to as SY5Y‐TauP301L‐EGFP cells. Stable expression of the inducible TauP301L‐EGFP construct was confirmed by fluorescence microscopy and Western blot after doxycycline induction.

SH‐SY5Y and SY5Y‐TauP301L‐EGFP cells were plated at 250,000 cells per well on Geltrex™‐coated culture plates (Gibco, # A1413201) and maintained in complete DMEM. After 24 h, the medium was replaced with CTS™ Neurobasal™ A Medium (Gibco, # A1371201) supplemented with 1× B‐27™ Supplement (ThermoFisher Scientific, #17504044), 10 μM L‐glutamine, and 1% Penicillin–Streptomycin (complete neurobasal medium). Cells were differentiated by treatment with 10 μM all‐trans‐retinoic acid (RA; Sigma‐Aldrich, # R2625) for 72 h. The differentiation was continued in complete neurobasal media supplemented with 10 μM RA and 50 ng/mL recombinant human brain‐derived neurotrophic factor (BDNF) (R&D Systems, #248‐BDB‐250) for another 72 h.

### 5.16 SH‐SY5Y seeding assay

Differentiated SY5Y‐TauP301L‐EGFP cells were pre‐treated with 0.5 μg/mL doxycycline (Sigma‐Aldrich, # D3447) for 24 h to induce the expression of TauP301L‐EGFP. Doxycycline was maintained in the cell culture media throughout the seeding assay. Cells were transduced with tau aggregation reaction products, standardized to 100 nM tau peptide. Transduction mixtures were prepared by combining the tau aggregation reaction products with TurboFect™ Transfection Reagent (Thermo Fisher Scientific Inc.) in Opti‐MEM™. The mixture was briefly vortexed and incubated at room temperature for 15 min before adding to cells. After 72 h, cells were processed for Triton X‐100 fractionation and western blot analysis, as described below.

### 5.17 Triton X‐100 fractionation and western blotting

Triton X‐100 soluble and insoluble fractions were prepared as described previously with minor modifications (Annadurai, Malina, Malohlava, et al., 2022). SY5Y‐TauP301L‐EGFP cells were harvested in 1× Tris‐buffered saline (TBS) containing 0.05% Triton X‐100, supplemented with protease and phosphatase inhibitors (Roche, #04693116001 and #04906837001). After centrifugation at 1000 × *g* for 10 min at 4°C, the supernatant was ultracentrifuged at 24,400 × *g* for 1 h at 4°C to separate Triton X‐100 soluble and insoluble fractions. The insoluble fraction was resuspended in radioimmunoprecipitation assay (RIPA) buffer (Thermo Fisher Scientific, #89901) with protease and phosphatase inhibitors for further analysis.

Equal volumes of soluble and insoluble fractions were resolved by SDS‐PAGE and transferred onto nitrocellulose membranes using the Trans‐Blot Turbo Transfer System (Bio‐Rad). Membranes were blocked with 5% BSA in 1× tris‐buffered saline with Tween‐20 (TBST) (0.1% Tween® 20) for 2 h at room temperature, followed by incubation with primary antibodies including anti‐tau Tau‐5 (1:500 or 1:1000; Invitrogen, Cat. #AHB0042), anti‐phospho Ser262 tau (1:1000; Invitrogen, Cat. # OPA1‐03142), anti‐oligomeric tau T22 (1:1000; Merck Millipore, Cat. # ABN454), and anti‐glyceraldehyde‐3‐phosphate dehydrogenase (GAPDH) (1:4000; SCBT, Cat. # sc‐32233), either overnight at 4°C or for 1 h at room temperature. Membranes were then incubated with Alexa Fluor 488‐conjugated secondary antibody (1:2000; Invitrogen; Cat. #A21202) for 1–2 h at room temperature in the dark. Protein bands were visualized using a Gel Doc XR+ System (Bio‐Rad), and densitometry analysis was performed with NIH ImageJ software (RRID: SCR_003070).

Densitometric analysis of insoluble tau bands was performed by first normalizing total tau signal intensities to GAPDH levels measured in the corresponding soluble fractions. For phosphorylated tau (pSer262) and oligomeric tau (T22), band intensities in the insoluble fraction were normalized to GAPDH and then adjusted relative to the total amount of transfected TauP301L‐EGFP. This two‐step normalization allowed assessment of pathological tau conversion. All normalized values were subsequently expressed as fold changes relative to the mean signal of the buffer‐treated control group (cells not exposed to fibrils).

### 5.18 Statistical analysis

All statistical analyses were performed using GraphPad Prism Software. Data are presented as mean ± standard error of the mean (SEM) unless otherwise specified, and *p* < 0.05 was considered statistically significant. Plots for SPR TCK experiments were generated using OriginPro version 8.5 (OriginLab Corporation, Northampton, MA).

## AUTHOR CONTRIBUTIONS

**Alladi Charanraj Goud:** Methodology; investigation; formal analysis; visualization. **Ihor Kozlov:** Methodology; investigation; visualization; formal analysis. **Patricie Skoupilová:** Methodology; investigation. **Lukáš Malina:** Methodology; investigation. **Sudeep Roy:** Methodology; investigation; visualization; formal analysis. **Viswanath Das:** Conceptualization; formal analysis; visualization; funding acquisition; supervision; writing – review and editing; writing – original draft.

## CONFLICT OF INTEREST STATEMENT

The authors declare no financial interests/personal relationships that may be considered potential competing interests.

## Supporting information


**Data S1.** The Supporting Information includes UV–Vis and SDS‐PAGE validation of tau monomers, replicate‐wise aggregation kinetics with sigmoidal fitting, fluorescence interference controls, AlphaFold2 structural models, protein–ligand interaction graphs, molecular dynamics simulations, ThT‐stained aggregate imaging, Western blot analyses of soluble and insoluble tau, full‐length blot images, and compound cytotoxicity assays. Docking and MM/GBSA data are provided in Tables S1 and S2.

## Data Availability

The data that support the findings of this study, including MM‐GBSA binding free energy calculations (Supporting Information Dataset S1), residue decomposition analysis (Supporting Information Dataset S2), and the complete computational methods for monomeric tau modeling, comprising structure preparation, docking protocols, scoring criteria, and molecular dynamics simulations, are openly available on Zenodo at https://doi.org/10.5281/zenodo.15492033. Any remaining data supporting the findings of this study are included within the article and its Supporting Information S1.

## References

[pro70240-bib-0001] Al Rihani SB , Darakjian LI , Kaddoumi A . Oleocanthal‐rich extra‐virgin olive oil restores the blood–brain barrier function through NLRP3 inflammasome inhibition simultaneously with autophagy induction in TgSwDI mice. ACS Chem Neurosci. 2019;10:3543–3554. 31244050 10.1021/acschemneuro.9b00175 PMC6703911

[pro70240-bib-0002] Annadurai N , Malina L , Malohlava J , Hajdúch M , Das V . Tau R2 and R3 are essential regions for tau aggregation, seeding and propagation. Biochimie. 2022;200:79–86. 35623497 10.1016/j.biochi.2022.05.013

[pro70240-bib-0003] Annadurai N , Malina L , Salmona M , Diomede L , Bastone A , Cagnotto A , et al. Antitumour drugs targeting tau R3 VQIVYK and Cys322 prevent seeding of endogenous tau aggregates by exogenous seeds. FEBS J. 2022;289:1929–1949. 34743390 10.1111/febs.16270

[pro70240-bib-0004] Aoyagi H , Hasegawa M , Tamaoka A . Fibrillogenic nuclei composed of P301L mutant tau induce elongation of P301L tau but not wild‐type tau. J Biol Chem. 2007;282:20309–20318. 17526496 10.1074/jbc.M611876200

[pro70240-bib-0005] Bloom GS . Amyloid‐β and tau: the trigger and bullet in Alzheimer disease pathogenesis. JAMA Neurol. 2014;71:505–508. 24493463 10.1001/jamaneurol.2013.5847 PMC12908160

[pro70240-bib-0006] Chen D , Bali S , Singh R , Wosztyl A , Mullapudi V , Vaquer‐Alicea J , et al. FTD‐tau S320F mutation stabilizes local structure and allosterically promotes amyloid motif‐dependent aggregation. Nat Commun. 2023;14:1625. 36959205 10.1038/s41467-023-37274-6 PMC10036635

[pro70240-bib-0007] Chen D , Drombosky KW , Hou Z , Sari L , Kashmer OM , Ryder BD , et al. Tau local structure shields an amyloid‐forming motif and controls aggregation propensity. Nat Commun. 2019;10:2493. 31175300 10.1038/s41467-019-10355-1 PMC6555816

[pro70240-bib-0008] Cino EA , Choy W‐Y , Karttunen M . Comparison of secondary structure formation using 10 different force fields in microsecond molecular dynamics simulations. J Chem Theory Comput. 2012;8:2725–2740. 22904695 10.1021/ct300323g PMC3419458

[pro70240-bib-0009] DeVos SL , Corjuc BT , Oakley DH , Nobuhara CK , Bannon RN , Chase A , et al. Synaptic tau seeding precedes tau pathology in human Alzheimer's disease brain. Front Neurosci. 2018;12:267. 29740275 10.3389/fnins.2018.00267 PMC5928393

[pro70240-bib-0010] DeVos SL , Miller RL , Schoch KM , Holmes BB , Kebodeaux CS , Wegener AJ , et al. Tau reduction prevents neuronal loss and reverses pathological tau deposition and seeding in mice with tauopathy. Sci Transl Med. 2017;9:eaag0481. 28123067 10.1126/scitranslmed.aag0481 PMC5792300

[pro70240-bib-0011] Ekman S , Flower R , Mahler S , Gould A , Barnard RT , Hyland C , et al. In silico molecular dynamics of human glycophorin a (GPA) extracellular structure. Ann Blood. 2021;6:11.

[pro70240-bib-0012] Friesner RA , Murphy RB , Repasky MP , Frye LL , Greenwood JR , Halgren TA , et al. Extra precision glide: docking and scoring incorporating a model of hydrophobic enclosure for protein−ligand complexes. J Med Chem. 2006;49:6177–6196. 17034125 10.1021/jm051256o

[pro70240-bib-0013] Gao Y‐L , Wang N , Sun F‐R , Cao X‐P , Zhang W , Yu J‐T . Tau in neurodegenerative disease. Ann Transl Med. 2018;6:175. 29951497 10.21037/atm.2018.04.23 PMC5994507

[pro70240-bib-0014] Genheden S , Ryde U . The MM/PBSA and MM/GBSA methods to estimate ligand‐binding affinities. Expert Opin Drug Discovery. 2015;10:449–461. 10.1517/17460441.2015.1032936 PMC4487606 25835573

[pro70240-bib-0015] Holmes BB , Furman JL , Mahan TE , Yamasaki TR , Mirbaha H , Eades WC , et al. Proteopathic tau seeding predicts tauopathy in vivo. Proc Natl Acad Sci U S A. 2014;111:E4376. 25261551 10.1073/pnas.1411649111 PMC4205609

[pro70240-bib-0016] Hong M , Zhukareva V , Vogelsberg‐Ragaglia V , Wszolek Z , Reed L , Miller BI , et al. Mutation‐specific functional impairments in distinct tau isoforms of hereditary FTDP‐17. Science. 1998;282:1914–1917. 9836646 10.1126/science.282.5395.1914

[pro70240-bib-0017] Hutton M , Lendon CL , Rizzu P , Baker M , Froelich S , Houlden H , et al. Association of missense and 5′‐splice‐site mutations in tau with the inherited dementia FTDP‐17. Nature. 1998;393:702–705. 9641683 10.1038/31508

[pro70240-bib-0018] Li W , Sperry JB , Crowe A , Trojanowski JQ , Smith AB , Lee VM‐Y . Inhibition of tau fibrillization by oleocanthal via reaction with the amino groups of tau. J Neurochem. 2009;110:1339–1351. 19549281 10.1111/j.1471-4159.2009.06224.x PMC2758489

[pro70240-bib-0019] Monteiro KLC , Mendonça T , de Aquino E , da Silva‐Júnior F . Natural compounds as inhibitors of aβ peptide and tau aggregation. CNS Neurol Disord Drug Targets. 2024;23:1234–1250. 38018200 10.2174/0118715273273539231114095300

[pro70240-bib-0020] Monti MC , Margarucci L , Riccio R , Casapullo A . Modulation of tau protein fibrillization by oleocanthal. J Nat Prod. 2012;75:1584–1588. 22988908 10.1021/np300384h

[pro70240-bib-0021] Monti MC , Margarucci L , Tosco A , Riccio R , Casapullo A . New insights on the interaction mechanism between tau protein and oleocanthal, an extra‐virgin olive‐oil bioactive component. Food Funct. 2011;2:423–428. 21894330 10.1039/c1fo10064e

[pro70240-bib-0022] Pounot K , Piersson C , Goring AK , Rosu F , Gabelica V , Weik M , et al. Mutations in tau protein promote aggregation by favoring extended conformations. JACS Au. 2024;4:92–100. 38274251 10.1021/jacsau.3c00550 PMC10806773

[pro70240-bib-0023] Schrödinger . Schrödinger Release 2025‐1: LigPrep. New York: Schrödinger, LLC; 2025a.

[pro70240-bib-0024] Schrödinger . Schrödinger Release 2025‐1: induced fit docking protocol; Glide. New York: Schrödinger, LLC, 2024; Prime, New York: Schrödinger, LLC; 2025b.

[pro70240-bib-0025] Schrödinger . Schrödinger Release 2025‐1: Glide. New York: Schrödinger, LLC; 2025c.

[pro70240-bib-0026] Schrödinger . Schrödinger Release 2025‐1: Desmond molecular dynamics system, D. E. Shaw Research, New York, 2024. Maestro‐Desmond interoperability tools. New York: Schrödinger, LLC; 2025d.

[pro70240-bib-0027] Schrödinger . Schrödinger Release 2025‐1: Protein Preparation Wizard; Epik. New York: Schrödinger, LLC; Impact. New York: Schrödinger, LLC; Prime. New York: Schrödinger, LLC; 2025e.

[pro70240-bib-0028] Seidler PM , Boyer DR , Murray KA , Yang TP , Bentzel M , Sawaya MR , et al. Structure‐based inhibitors halt prion‐like seeding by Alzheimer's disease‐ and tauopathy‐derived brain tissue samples. J Biol Chem. 2019;294:16451–16464. 31537646 10.1074/jbc.RA119.009688 PMC6827308

[pro70240-bib-0029] Soeda Y , Saito M , Maeda S , Ishida K , Nakamura A , Kojima S , et al. Methylene blue inhibits formation of tau fibrils but not of granular tau oligomers: a plausible key to understanding failure of a clinical trial for Alzheimer's disease. J Alzheimer's Dis. 2019;68:1677–1686. 30909223 10.3233/JAD-181001

[pro70240-bib-0030] Soeda Y , Takashima A . New insights into drug discovery targeting tau protein. Front Mol Neurosci. 2020;13:590896. 33343298 10.3389/fnmol.2020.590896 PMC7744460

[pro70240-bib-0031] Spillantini MG , Goedert M . Tau pathology and neurodegeneration. Lancet Neurol. 2013;12:609–622. 23684085 10.1016/S1474-4422(13)70090-5

[pro70240-bib-0032] Spillantini MG , Murrell JR , Goedert M , Farlow MR , Klug A , Ghetti B . Mutation in the tau gene in familial multiple system tauopathy with presenile dementia. Proc Natl Acad Sci U S A. 1998;95:7737–7741. 9636220 10.1073/pnas.95.13.7737 PMC22742

[pro70240-bib-0033] Spittaels K , Van den Haute C , Van Dorpe J , Bruynseels K , Vandezande K , Laenen I , et al. Prominent axonopathy in the brain and spinal cord of transgenic mice overexpressing four‐repeat human tau protein. Am J Pathol. 1999;155:2153–2165. 10595944 10.1016/S0002-9440(10)65533-2 PMC1866931

[pro70240-bib-0034] Strang KH , Golde TE , Giasson BI . MAPT mutations, tauopathy, and mechanisms of neurodegeneration. Lab Investig. 2019;99:912–928. 30742061 10.1038/s41374-019-0197-x PMC7289372

[pro70240-bib-0035] Sun KT , Patel T , Kang S‐G , Yarahmady A , Srinivasan M , Julien O , et al. Disease‐associated mutations in tau encode for changes in aggregate structure conformation. ACS Chem Nerosci. 2023;14:4282–4297. 10.1021/acschemneuro.3c00422 PMC10741665 38054595

[pro70240-bib-0036] Viswanathan GK , Shwartz D , Losev Y , Arad E , Shemesh C , Pichinuk E , et al. Purpurin modulates tau‐derived VQIVYK fibrillization and ameliorates Alzheimer's disease‐like symptoms in animal model. Cell Mol Life Sci. 2020;77:2795–2813. 31562564 10.1007/s00018-019-03312-0 PMC11104911

[pro70240-bib-0037] von Bergen M , Barghorn S , Li L , Marx A , Biernat J , Mandelkow E‐M , et al. Mutations of tau protein in frontotemporal dementia promote aggregation of paired helical filaments by enhancing local β‐structure. J Biol Chem. 2001;276:48165–48174. 11606569 10.1074/jbc.M105196200

[pro70240-bib-0038] Wang X , Friesen E , Müller I , Lemieux M , Dukart R , Maia IB , et al. Rapid generation of human neuronal cell models enabling inducible expression of proteins‐of‐interest for functional studies. Bio Protoc. 2020;10:e3615. 10.21769/BioProtoc.3615 PMC7842556 33659578

[pro70240-bib-0039] Xia Y , Sorrentino ZA , Kim JD , Strang KH , Riffe CJ , Giasson BI . Impaired tau–microtubule interactions are prevalent among pathogenic tau variants arising from missense mutations. J Biol Chem. 2019;294:18488–18503. 31653695 10.1074/jbc.RA119.010178 PMC6885647

[pro70240-bib-0040] Yao Q‐Q , Hong L , Wu S , Perrett S . Distinct microscopic mechanisms for the accelerated aggregation of pathogenic tau mutants revealed by kinetic analysis. Phys Chem Chem Phys. 2020;22:7241–7249. 32207466 10.1039/c9cp06083a

[pro70240-bib-0041] Yao T‐M , Tomoo K , Ishida T , Hasegawa H , Sasaki M , Taniguchi T . Aggregation analysis of the microtubule binding domain in tau protein by spectroscopic methods. J Biochem. 2003;134:91–99. 12944375 10.1093/jb/mvg116

[pro70240-bib-0042] Young AL , Bocchetta M , Russell LL , Convery RS , Peakman G , Todd E , et al. Characterizing the clinical features and atrophy patterns of MAPT‐related frontotemporal dementia with disease progression modeling. Neurology. 2021;97:e941–e952. 34158384 10.1212/WNL.0000000000012410 PMC8408507

[pro70240-bib-0043] Zhu L , Qian Z . Recent studies of atomic‐resolution structures of tau protein and structure‐based inhibitors. Quant Biol. 2022;10:17–34.

