# Supplementary material for: Structural and functional insights into the selective inhibition of mutant tau aggregation by purpurin and oleocanthal in frontotemporal dementia
Source: Protein Sci. 2025 Aug 27;34(9):e70240. doi: 10.1002/pro.70240 (PMC12381781; doi:10.1002/pro.70240)
Supplement: Supplementary file 1 — Data S1. The Supporting Information includes UV–Vis and SDS‐PAGE validation of tau monomers, replicate‐wise aggregation kinetics with sigmoidal fitting, fluorescence interference controls, AlphaFold2 structural models, protein–ligand interaction graphs, molecular dynamics simulations, ThT‐stained aggregate imaging, Western blot analyses of soluble and insoluble tau, full‐length blot images, and compound cytotoxicity assays. Docking and MM/GBSA data are provided in Tables S1 and S2. [file PRO-34-e70240-s001.docx]

**Structural and Functional Insights into the Selective Inhibition of Mutant Tau Aggregation by Purpurin and Oleocanthal in Frontotemporal Dementia**

Alladi Charanraj Goud^1^, Ihor Kozlov^1^, Patricie Skoupilová^1^, Lukáš Malina^1,2^, Sudeep Roy^3^, Viswanath Das ^1,4^

^1^ Institute of Molecular and Translational Medicine, Faculty of Medicine and Dentistry, Palacký University and University Hospital Olomouc, Hněvotínská 1333/5, 779 00 Olomouc, Czech Republic

^2^ Department of Medical Biophysics, Faculty of Medicine and Dentistry, Palacký University Olomouc, Hněvotínská 3, 775 15 Olomouc, Czech Republic

^3^ Department of Biomedical Engineering, Faculty of Electrical Engineering and Communication, Brno University of Technology, Technická 12, Brno, 616 00, Czech Republic

^4^ Institute of Molecular and Translational Medicine, Czech Advanced Technologies and Research Institute, Palacký University Olomouc, Křížkovského 511/8, 779 00, Olomouc, Czech Republic

| \| **Table of content** \| **Page** \| \| --- \| --- \| \| Figure S1. UV-Vis analysis of tau peptide monomers \| S2-S3 \| \| Figure S2. SDS-PAGE validation of monomeric tau peptides \| S3 \| \| Figure S3. Sigmoidal fittings of WT and mutant peptides \| S4 \| \| Figure S4. Control fluorescence measurements of ThT, purpurin, and oleocanthal \| S4 \| \| Figure S5. Sigmoidal fittings of R2R3 (WT) with PUR and OLC \| S5 \| \| Figure S6. Sigmoidal fittings of R2R3 (P301L) with PUR and OLC \| S6 \| \| Figure S7. Sigmoidal fittings of R2R3 (V287I) with PUR and OLC \| S7 \| \| Figure S8. Sigmoidal fittings of R2R3 (N279K) with PUR and OLC \| S8 \| \| Figure S9. AlphaFold2 modeling of WT and P301L peptides \| S9 \| \| Figure S10. Ligand-binding maps with WT and P301L monomers \| S10 \| \| Figure S11. ThT-stained imaging of aggregates (corresponding to Figure 3) \| S11 \| \| Figure S12. ThT-stained aggregates from post-treatment samples (corresponding to Fig. 4) \| S12 \| \| Figure S13. Inhibition of elongation in V287I and N279K \| S13 \| \| Figure S14. MD simulation of WT filament with PUR and OLC \| S14 \| \| Figure S15. MD simulation of P301L filament with PUR and OLC \| S15 \| \| Figure S16. Cytotoxicity assays for PUR and OLC in biosensor and CCRF-CEM cells \| S16 \| \| Figure S17. Effect of PUR and OLC only on tau seeding \| S17 \| \| Figure S18. Western blot validation of high molecular weight tau in SY5Y cells \| S18 \| \| Figure S19. Soluble tau analysis from SY5Y-P301L cells \| S19 \| \| Figure S20. Western blots of WT tau aggregation products on SY5Y cells \| S20 \| \| Figure S21. Full-length Western blot images for Figure 8 \| S21 \| \| Table S1. IFD scores and energy terms (WT and P301L) \| S22 \| \| Table S2. MM-GBSA energies (WT and P301L) \| S22 \| |
| --- | --- | --- | --- | --- | --- | --- | --- | --- | --- | --- | --- | --- | --- | --- | --- | --- | --- | --- | --- | --- | --- | --- | --- | --- | --- | --- | --- | --- | --- | --- | --- | --- | --- | --- | --- | --- | --- | --- | --- | --- | --- | --- | --- | --- | --- | --- | --- | --- |


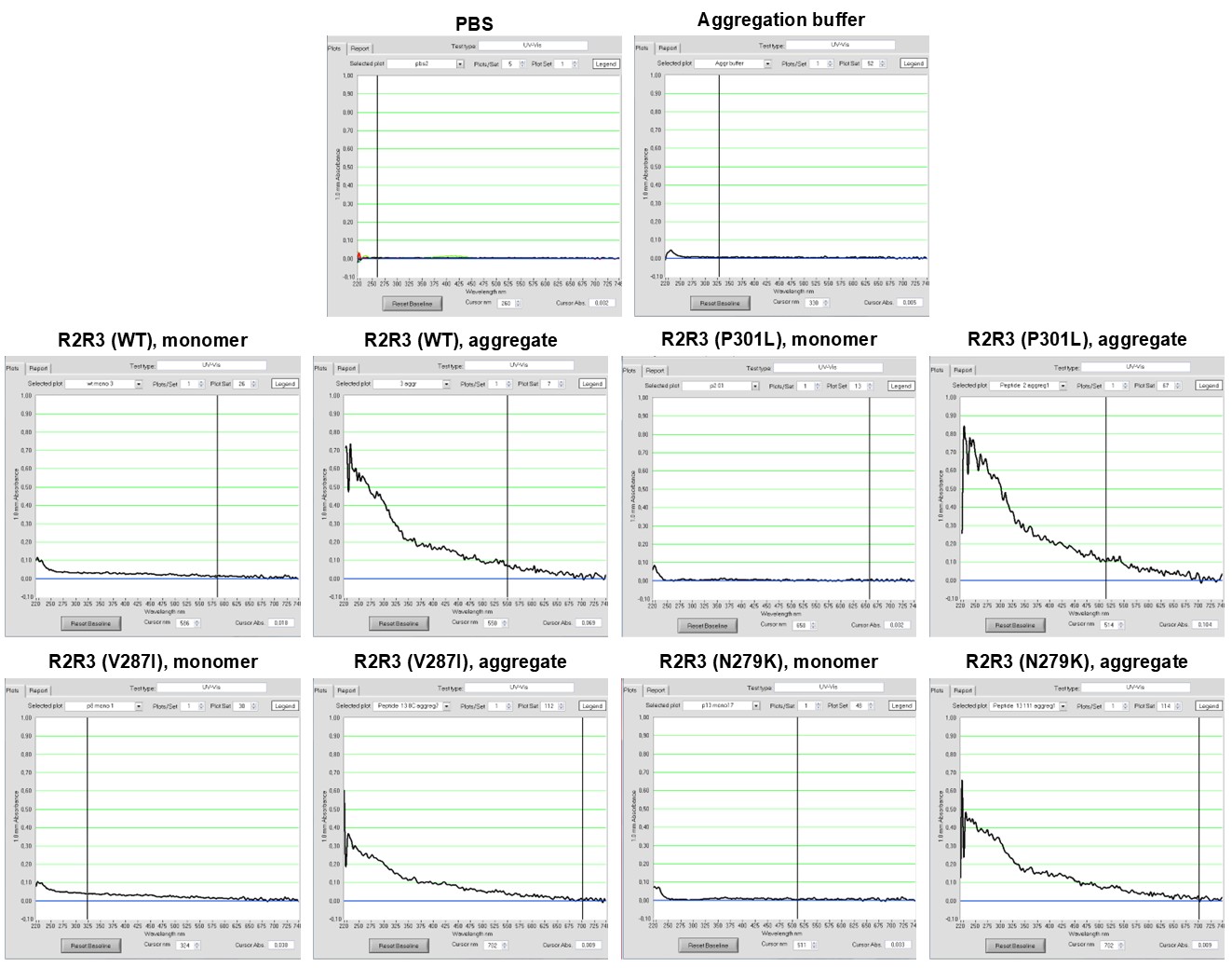
.

**Figure S1. UV-Vis spectral analysis of tau peptide samples confirms monomeric starting material and aggregation-associated light scattering.** UV-Vis spectra (220–750 nm) were recorded using 1 µL of PBS (a), aggregation buffer (b), freshly thawed monomeric peptide from a 154 µM stock (c), and peptide samples after 48 h of aggregation (d), using a NanoDrop 1000 spectrophotometer. Each sample was measured in triplicate and averaged; baseline correction was performed using RNase-free ddH₂O. The spectra of PBS, aggregation buffer, and monomeric peptide showed flat profiles with low absorbance, indicating optically clear solutions and absence of light-scattering species. In contrast, aggregated samples exhibited strong absorbance below 300 nm and a broad increase across the visible range, consistent with light scattering by fibrillar aggregates. These results confirm that the peptides were in a monomeric state at the start of the ThT assays and underwent aggregation during the incubation period.

**
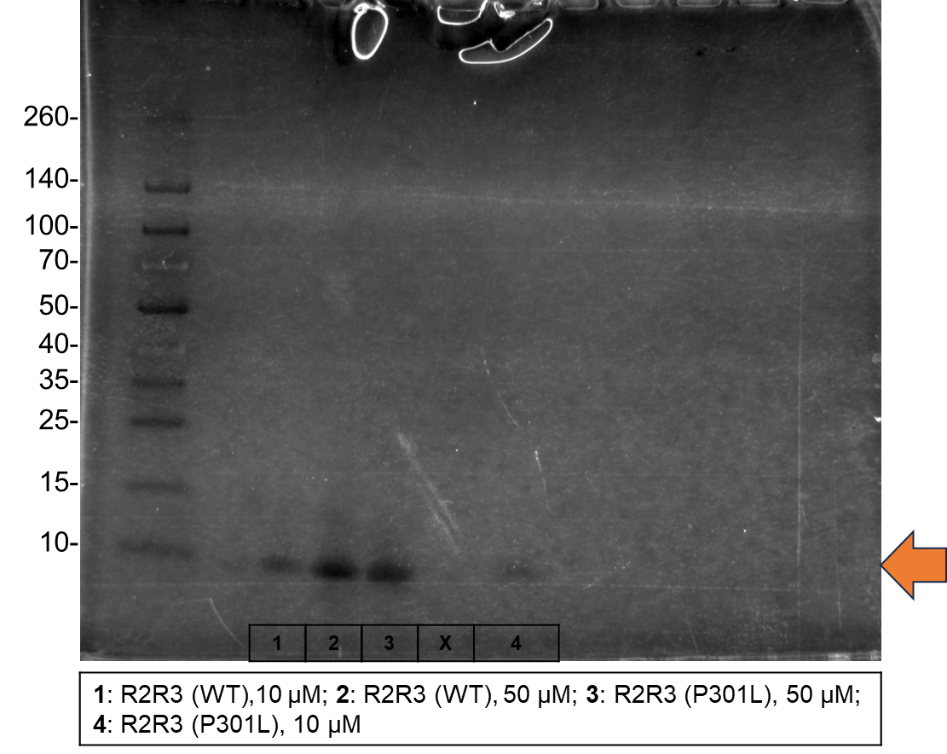
**

**Figure S2**. **Confirmation of monomeric tau peptides by SDS-PAGE.** WT and P301L R2R3 tau peptides were analyzed at 10 and 50 µM concentrations to confirm their monomeric state. Peptides were prepared from 154 µM stock solutions in Milli-Q water, centrifuged at 13,000 RPM for 1 min, and mixed with non-reducing 5× Laemmli sample buffer. SDS-PAGE was performed using 4-20% Mini-PROTEAN® TGX™ precast gels (Bio-Rad, #4561095). Gels were stained with Coomassie Brilliant Blue R-250 and destained with 40% methanol/10% acetic acid for 3 h. Gels were imaged using the ChemiDoc™ MP Imaging System (Bio-Rad). Monomeric tau (~10 kDa) appeared as a single band in each lane and is indicated by the orange arrow.

**
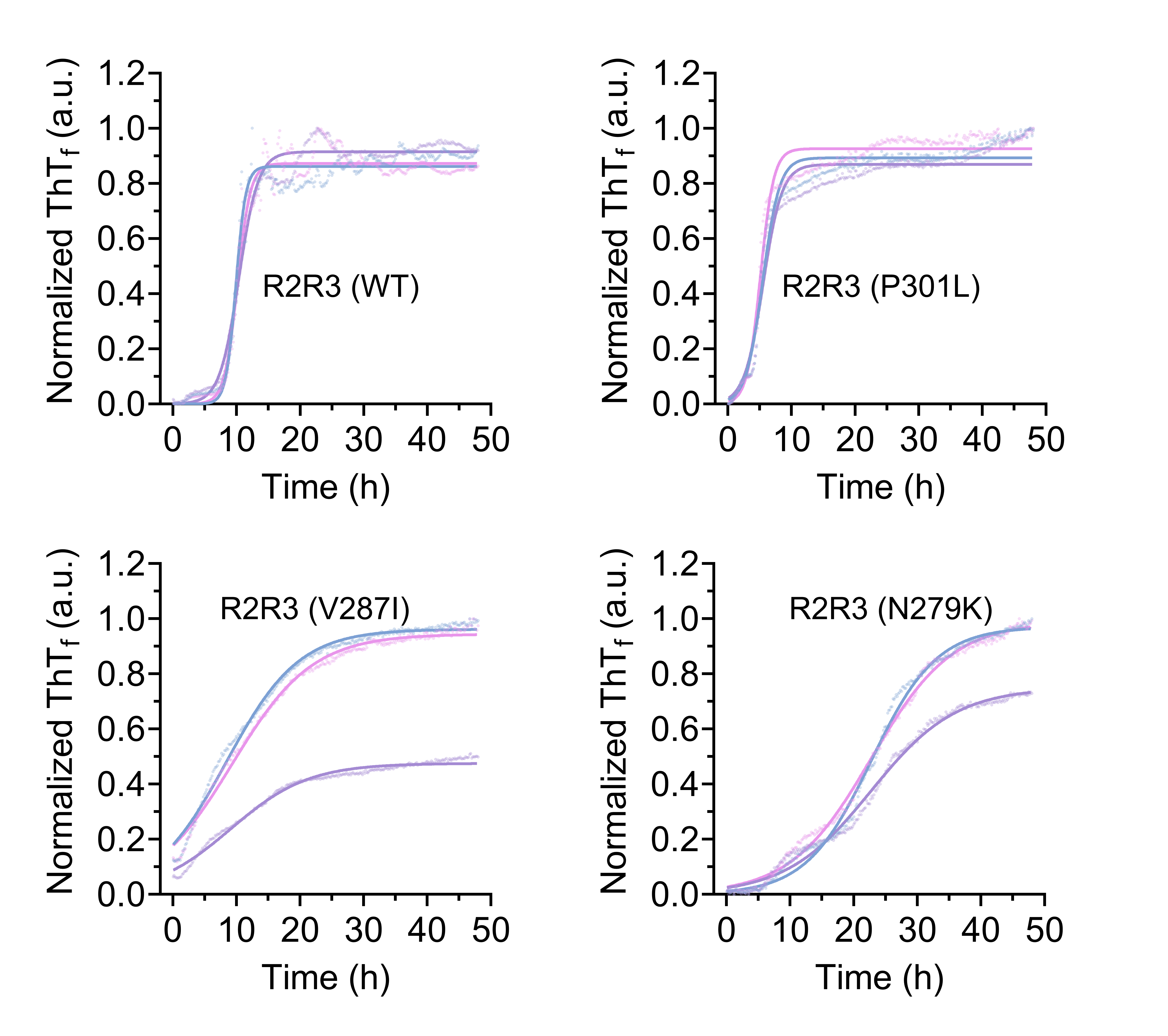
**

**Figure S3. Aggregation kinetics of WT and mutant R2R3 tau peptides with replicate-wise sigmoidal fitting.** ThT fluorescence curves from three replicates are shown for each peptide (WT, P301L, V287I, and N279K). ThT fluorescence data were fitted to a sigmoidal function (shown as dashed lines), and the V50 parameter from each fit was used as the aggregation halftime. Goodness-of-fit (R^2^) values were >0.95 across replicates. Aggregation halftimes derived from these fits were used for statistical analysis as reported in Figure 1c.

**Figure S4. Control fluorescence measurements of ThT, purpurin, and oleocanthal.** ThT (15 µM), purpurin (10 µM), and oleocanthal (10 µM) were incubated individually in the absence of tau peptides in the aggregation buffer for 48 h. Fluorescence was recorded using excitation/emission filters specific for ThT (λex: 460-490 nm, λem: 500-550 nm). The absence of fluorescence changes confirms that purpurin and oleocanthal do not interfere with ThT readings independently. Mean ± SEM (*n* = 3).


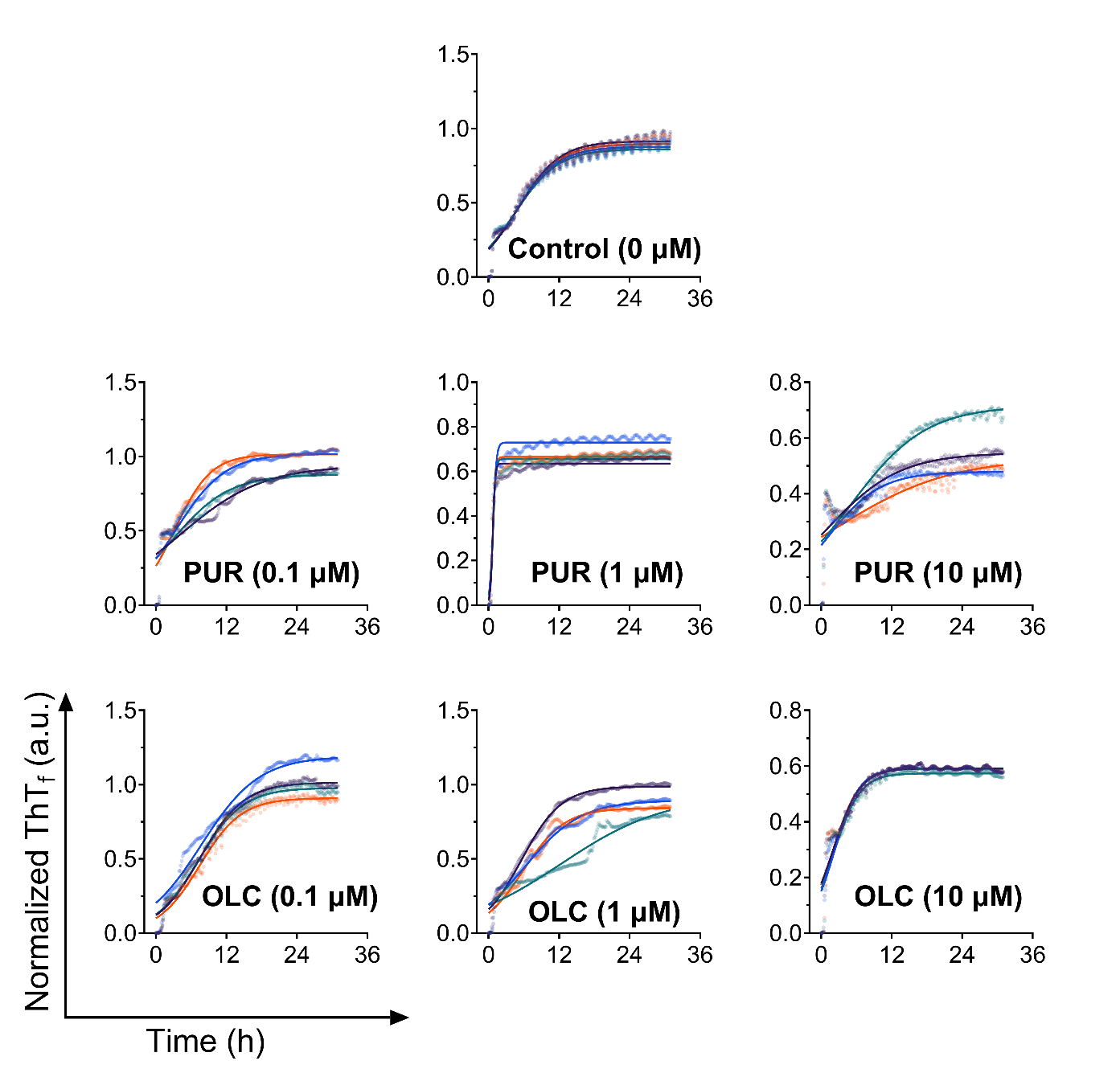
**Figure S5. Sigmoidal fitting of aggregation kinetics for R2R3 (WT) peptides treated with PUR or OLC.** Individual ThT fluorescence traces for four replicates are shown for each condition (control, PUR, or OLC at 0.1, 1, and 10 µM), with overlaid sigmoidal curve fits (colored lines). Aggregation halftimes (V50) were extracted for all conditions based on valid sigmoidal fits, including those with low-amplitude fluorescence.


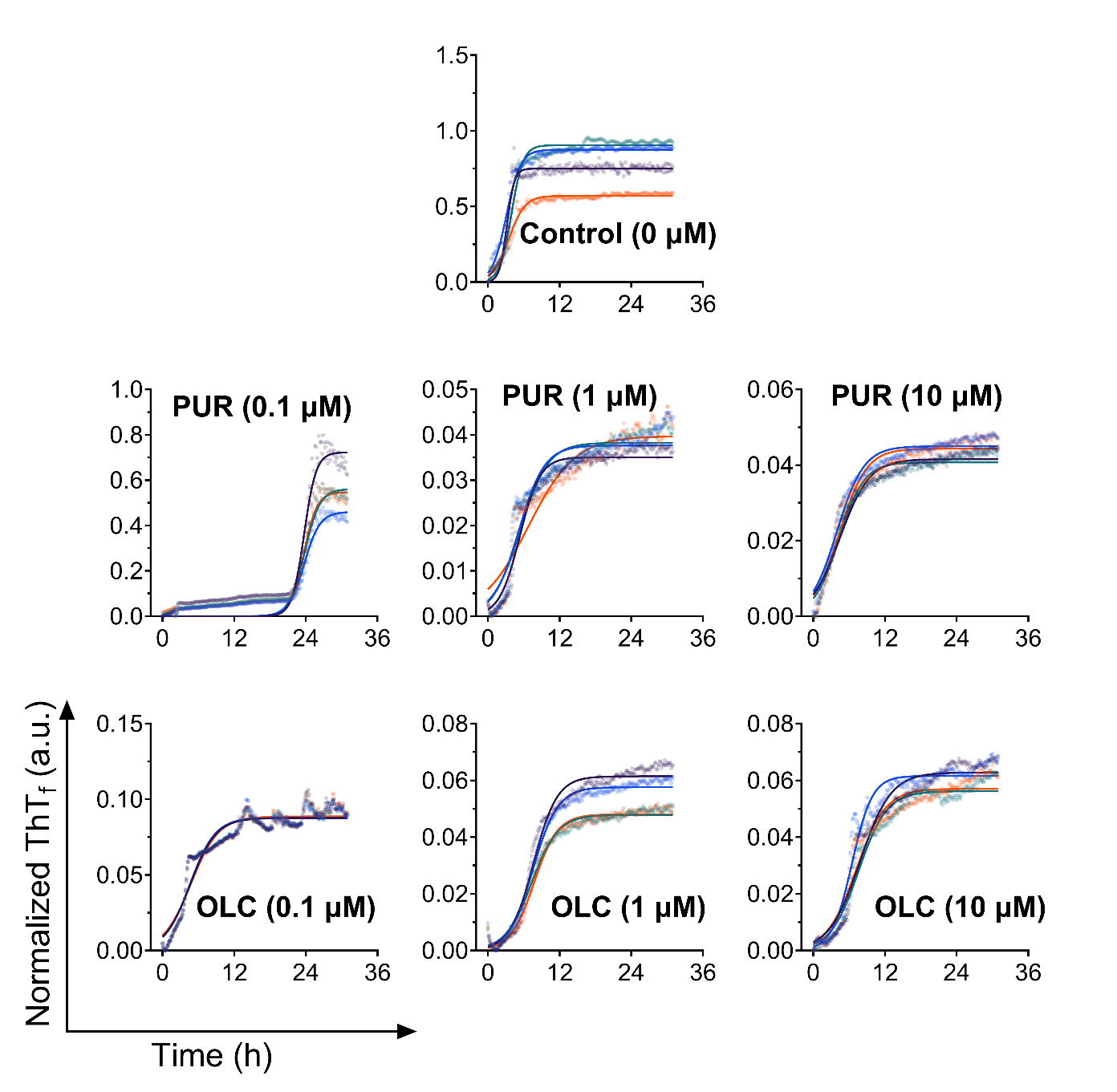


**Figure S6. Sigmoidal fitting of aggregation kinetics for R2R3 (P301L) peptides treated with PUR or OLC.** Individual ThT fluorescence traces for four replicates are shown for each condition (control, PUR, or OLC at 0.1, 1, and 10 µM), with overlaid sigmoidal curve fits (colored lines). Aggregation halftimes (V50) were extracted for all conditions based on valid fits, including low-amplitude traces where sigmoidal transitions were retained.


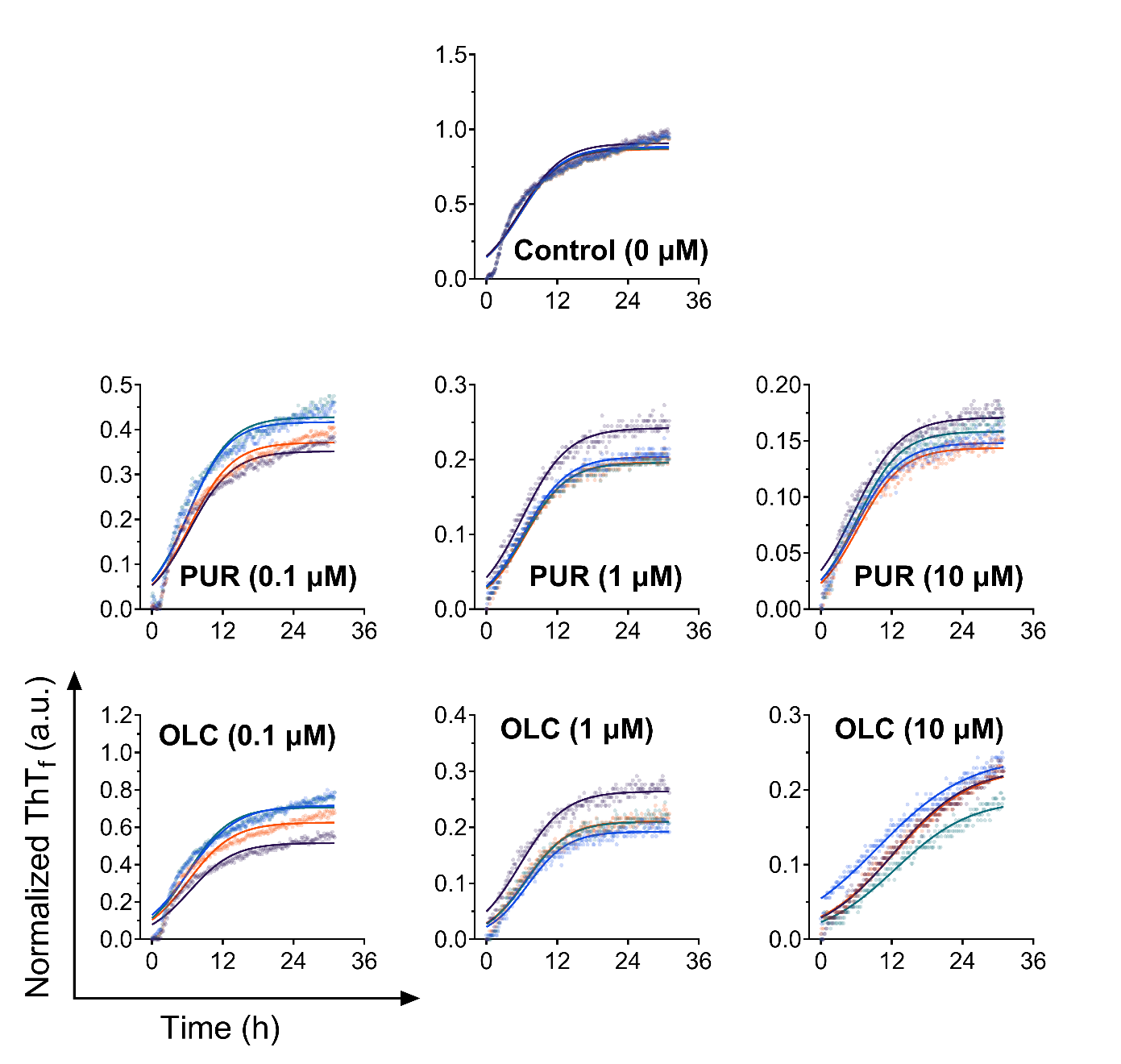


**Figure S7. Sigmoidal fitting of aggregation kinetics for R2R3 (V287I) peptides treated with PUR or OLC**. Individual ThT fluorescence traces for four replicates are shown for each condition (control, PUR, or OLC at 0.1, 1, and 10 µM), with overlaid sigmoidal curve fits (colored lines). Aggregation halftimes (V50) were extracted for all conditions based on valid sigmoidal fits, including those with low-amplitude fluorescence.


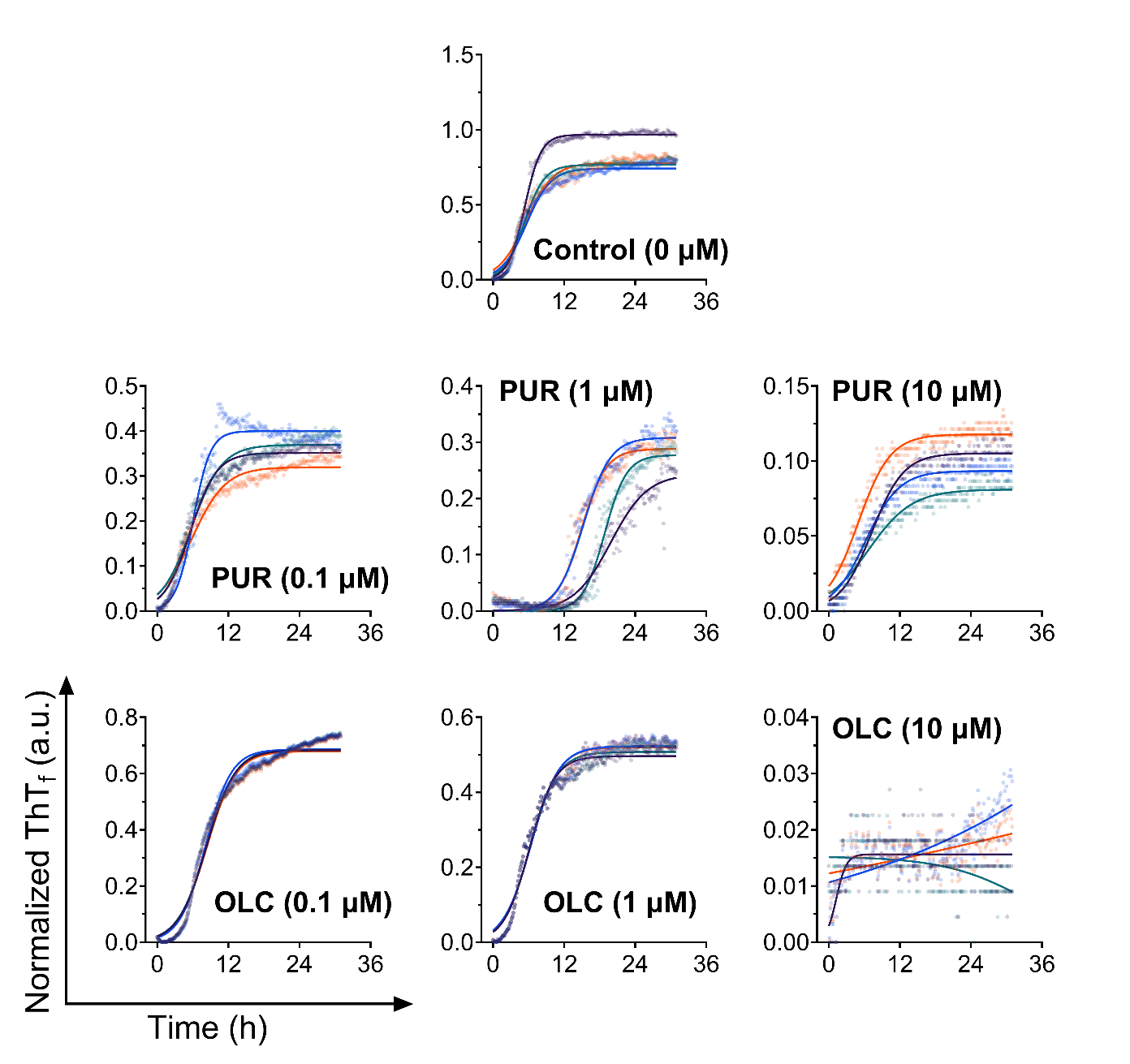


**Figure S8. Sigmoidal fitting of aggregation kinetics for R2R3 (N279K) peptides treated with PUR or OLC.** Individual ThT fluorescence traces for four replicates are shown for each condition (control, PUR, or OLC at 0.1, 1, and 10 µM), with overlaid sigmoidal curve fits (colored lines). Aggregation halftimes (V50) were extracted for all conditions with valid sigmoidal fits. No reliable fitting was obtained for the 10 µM OLC condition, and V50 is listed as ‘undefined’.

**
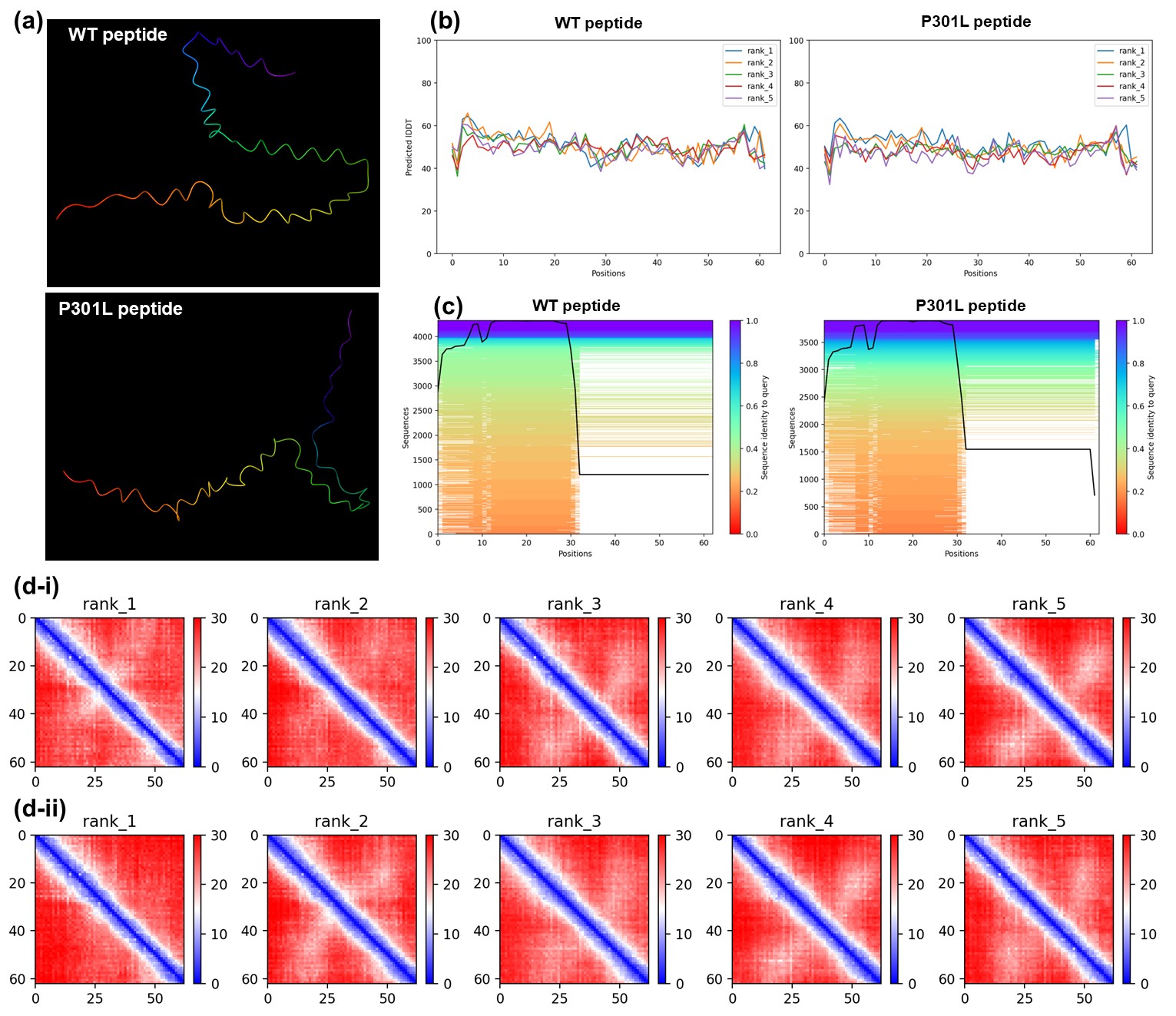
**

**Figure S9. AlphaFold2-based structural modeling and confidence assessment of WT and P301L tau peptide monomers.** (**a**) Structural models of WT and P301L tau peptide monomers, corresponding to the experimental sequences used in aggregation assays, were generated using AlphaFold2. (**b**) Predicted local distance difference test (pLDDT) scores for WT and P301L peptide models. Higher scores indicate greater confidence in local residue positioning. (**c**) Sequence coverage plots for WT and P301L tau peptide models, reflecting the relative prediction confidence across the sequence. (**d**) Predicted aligned error (PAE) maps for WT (**d-i**) and P301L (**d-ii**) tau peptide models. Blue blocks along the diagonal indicate higher confidence in local residue positioning, while red regions denote lower confidence in long-range residue relationships. AlphaFold2 modeling of tau peptide sequences yielded low overall pLDDT scores (~50-52) and high PAE values, indicating limited structural confidence and extensive positional uncertainty. Sequence coverage plots showed low-confidence predictions across most residues, consistent with the intrinsically disordered and aggregation-prone nature of the tau R2R3 region. These data support the expected structural flexibility of WT and P301L tau peptides and justify their use in peptide-level docking analyses. Detailed methodology is available in Zenodo at https://doi.org/10.5281/zenodo.15492033.


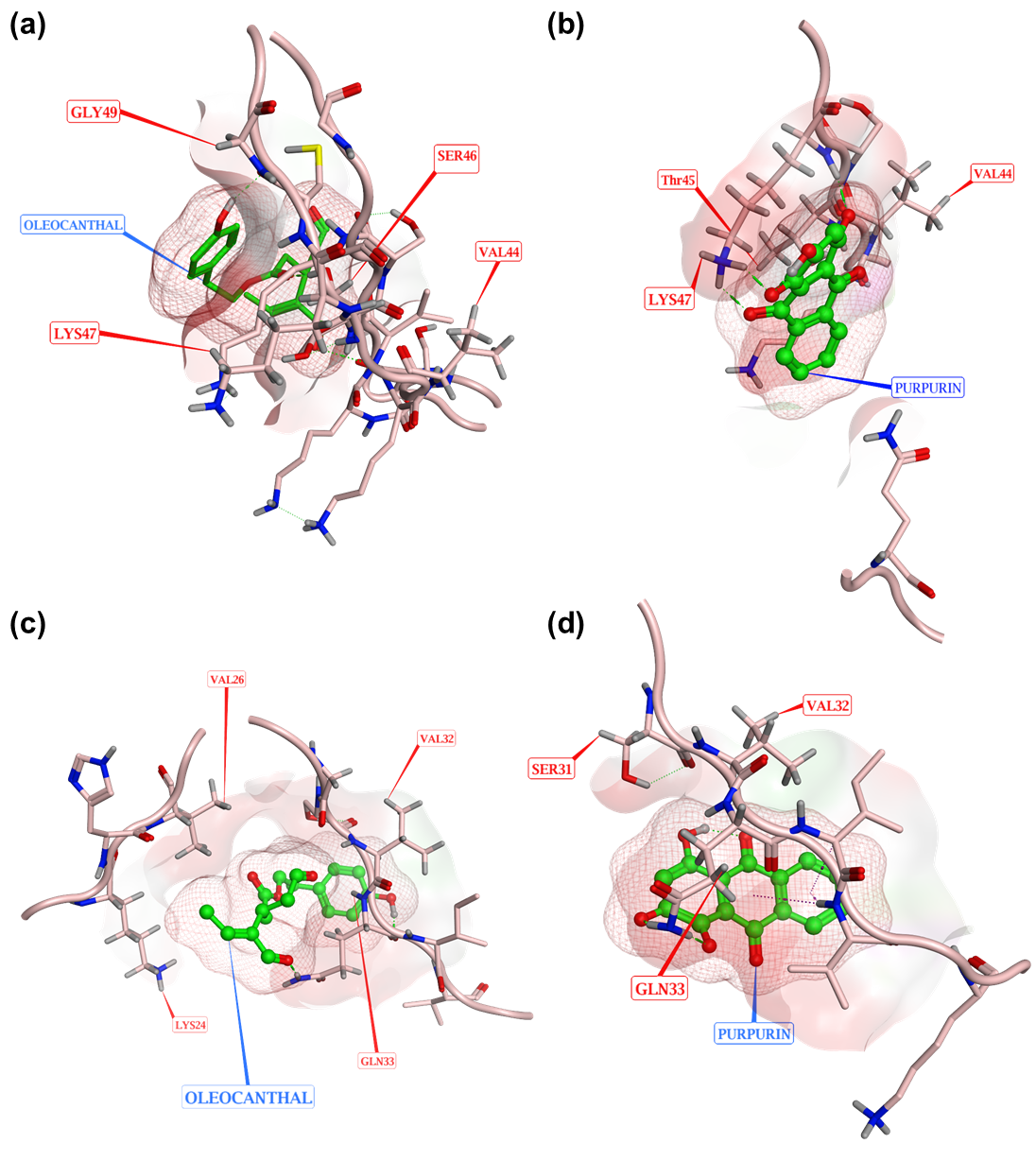


**Figure S10. Predicted ligand-binding interactions of PUR and OLC with monomeric WT and P301L tau peptides**. 2D interaction maps illustrate the hydrogen bonding patterns formed between each ligand and the AlphaFold2-predicted tau peptide models. In the WT model, OLC interacts with VAL44, SER46, and GLY49 (**a**), while PUR engages THR45 and LYS47 (**b**). In the P301L model, OLC forms hydrogen bonds with GLY30 and GLN33 (**c**), and PUR interacts with SER31 and GLN33 (**d**). These interactions reveal a shift in binding site location from mid-to-C-terminal residues in WT peptides to more N-terminal residues in P301L. This shift aligns with the more favorable docking scores and MM-GBSA binding free energies for P301L compared to WT for both ligands, as reported in Tables S1 and S2. Detailed methodology is available in Zenodo at https://doi.org/10.5281/zenodo.15492033.


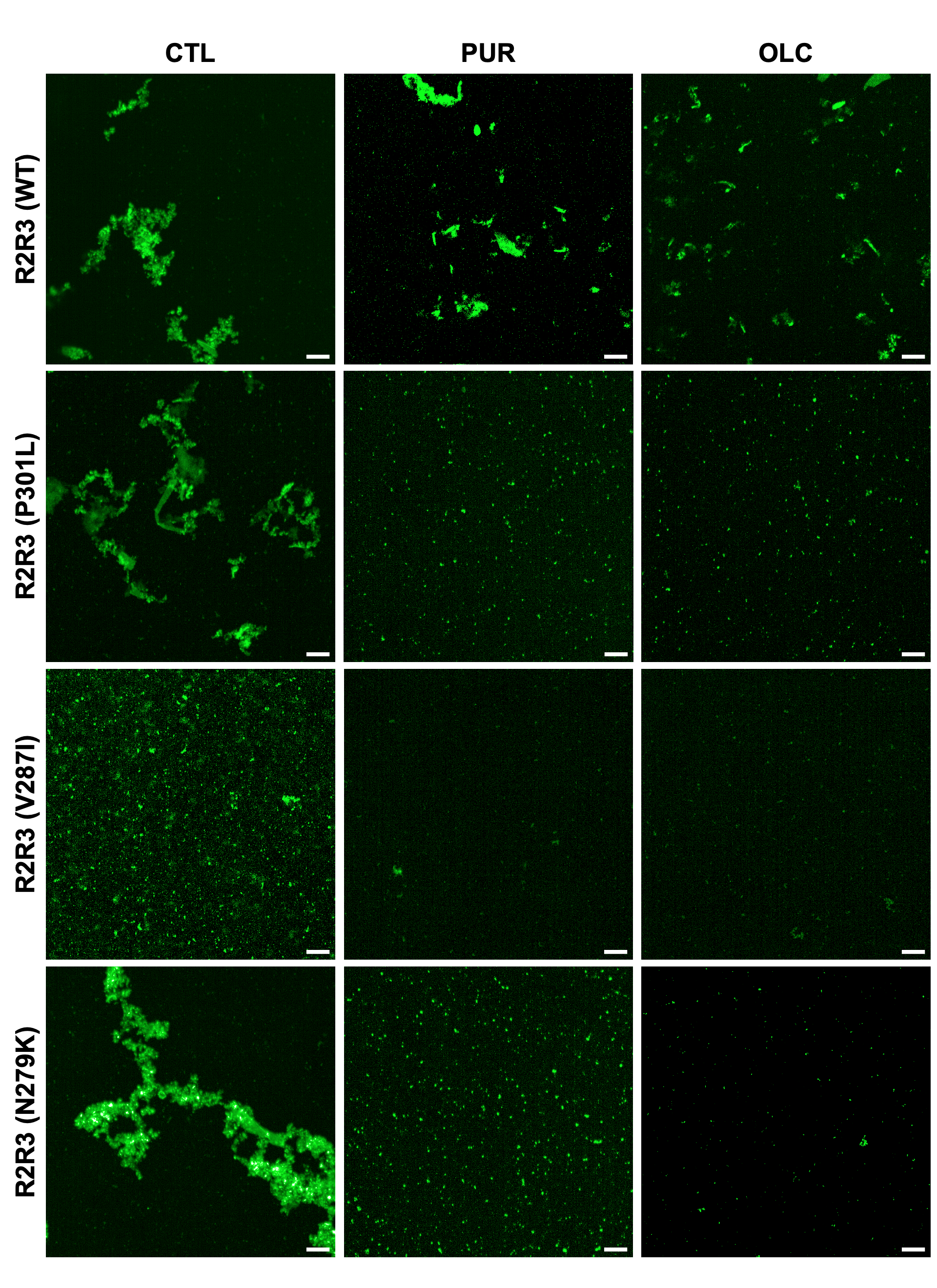


**Figure S1****1. Confocal imaging of ThT-stained aggregates formed in the presence of PUR and OLC (corresponding to Figure 3).** At the end of the ThT aggregation assay, reaction mixtures containing WT or mutant R2R3 tau peptides (P301L, V287I, N279K), incubated in the absence (CTL) or presence of 10 µM PUR or OLC, were collected from assay wells, embedded in 0.05% low-melting agarose, and imaged. ThT fluorescence was acquired using a Yokogawa Cell Voyager 7000S high-content imaging system equipped with a 20× objective (Ex: 460–490 nm, Em: 500–550 nm) and analyzed with Signals Image Artist (Revvity). CTL samples displayed intense, clustered ThT-positive fluorescence, reflecting robust accumulation of β-sheet-rich aggregates. In contrast, treatment with PUR or OLC led to a marked reduction in ThT signal in all three mutant peptides, with only sparse or undetectable aggregates. WT samples showed a moderate reduction in fluorescence intensity. These results are consistent with the ThT kinetic data in Fig. 3 and support the inhibitory activity of PUR and OLC against tau aggregation. Scale bar: 20 μm.


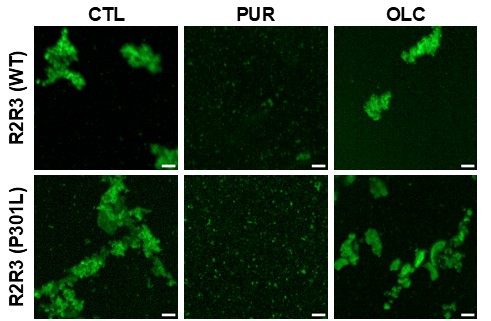


**Figure S12.** **Confocal imaging of ThT-stained aggregates from post-treatment samples in Figure 4.** R2R3 (WT and P301L) tau peptides were incubated for 24 h to allow initial aggregation, followed by the addition of PUR or OLC (10 µM). After a further 24 h, reaction mixtures from compound-treated wells were collected, embedded in 0.05% low-melting agarose, and processed for imaging as described in Figure S11. PUR- and OLC-treated samples exhibited either a complete absence or a marked reduction in ThT signal, consistent with inhibition of further aggregate accumulation. Scale bar: 50 μm.


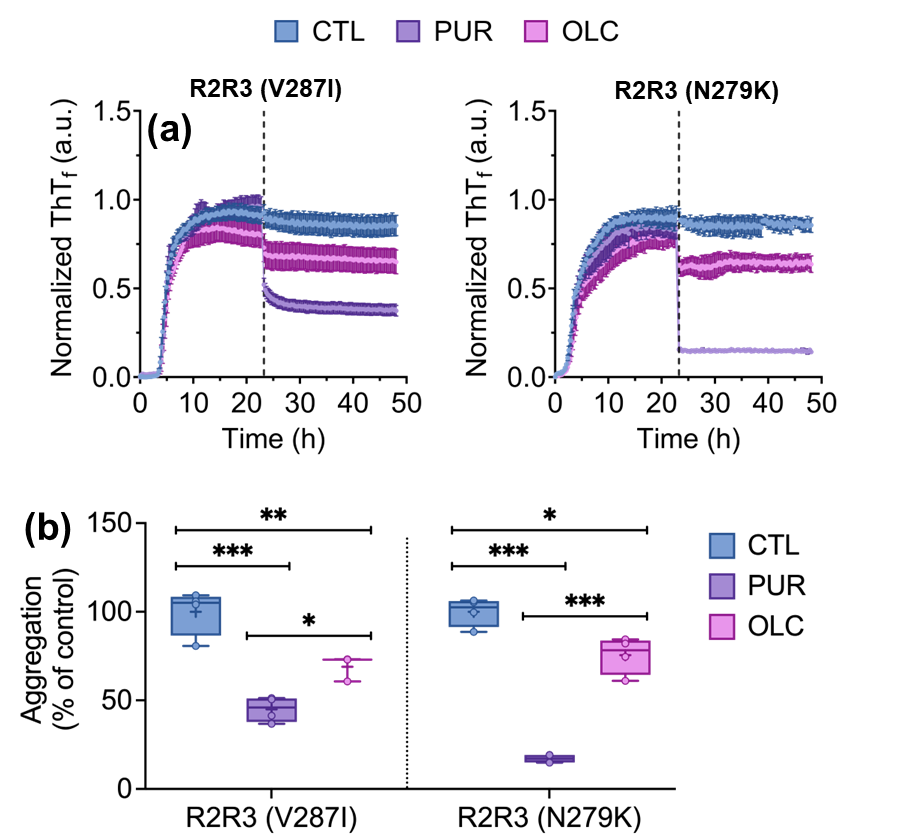


**Figure S13**. **PUR and OLC inhibit the elongation of preformed fibrils of V287I and N279K peptides**. (**a**) ThT fluorescence kinetics of pre-aggregated R2R3 (V287I and N279K ) tau peptides treated with PUR or OLC (10 µM). Peptides were first allowed to aggregate for 24 h, followed by compound addition and continued incubation for an additional 24-30 h. Vertical dashed lines indicate the time point of compound addition. Curves are normalized to each replicate’s maximum pre-treatment ThT fluorescence. Source data are provided in the Data Availability section. Mean ± SEM (*n* ≥ 3). (**b**) Aggregation levels post-treatment, expressed as a percentage of control (untreated fibrils), calculated from the AUC after compound addition. Mean ± SEM (n ≥ 3). ***P < 0.001, **P < 0.05, *P < 0.05, one-way ANOVA (Tukey’s post hoc test).


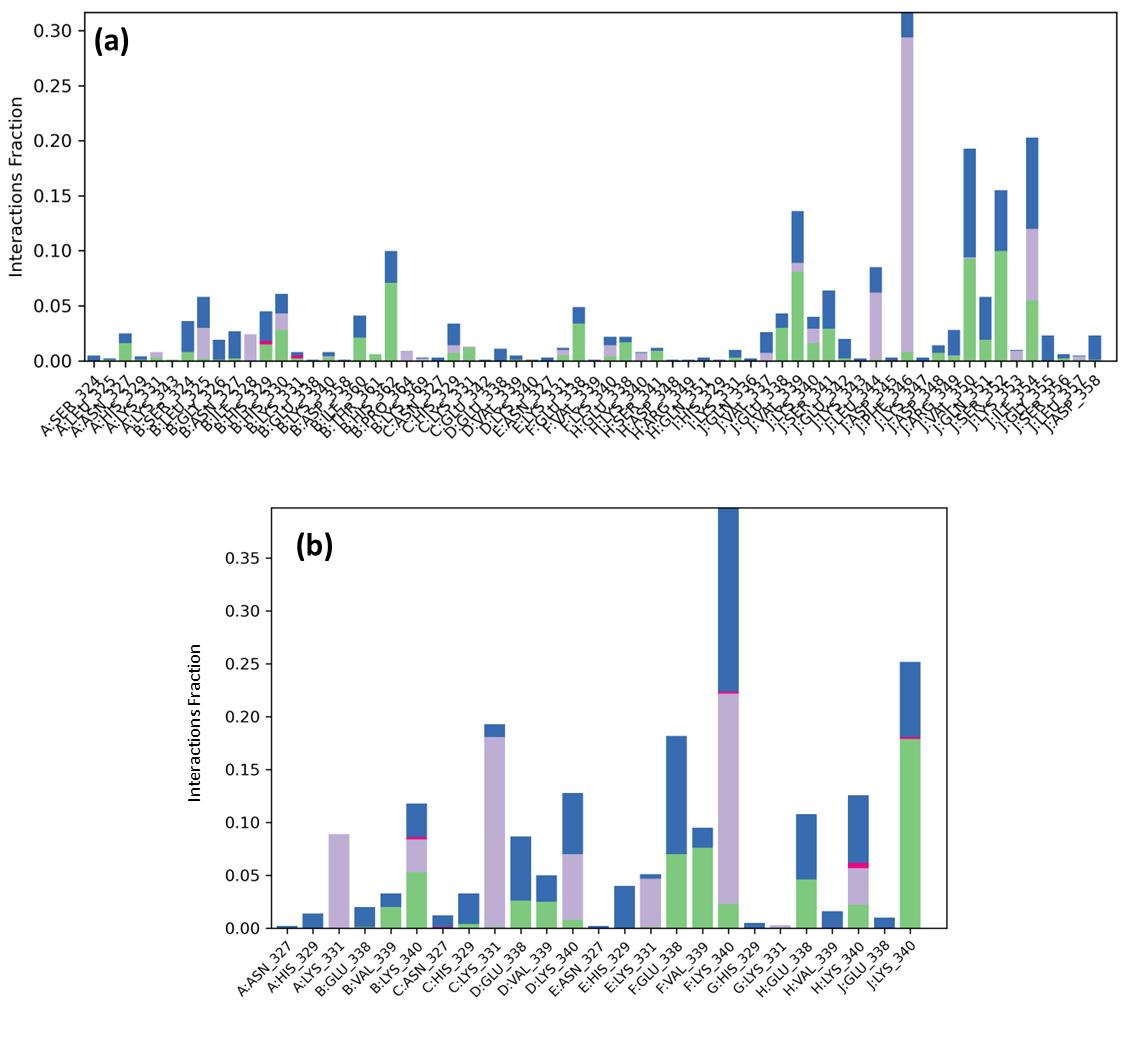


**Figure S14.** **Molecular simulation studies of wild tau filament with compounds**. A schematic representation of the interaction of 5O3L with the compounds (a) Oleocanthal and (b) Purpurin in molecular dynamics simulations. Interactions that occurred more than 30% of the simulation time in the selected trajectory (0.00 through 100 ns) are shown. These interactions can be categorized by the types of bonding, which are normalized over the course of the trajectory. Each interaction type contains more specific subtypes, which can be explored through the “Simulation Interaction Diagram” panel. These interactions (or “contacts”) are mainly categorized into three types and are represented in the above plot as hydrogen (green), hydrophobic (purple), and water bridge (blue) bonds. The docking-based interaction studies was carried out with the help of Schrödinger Release 2025-1. The molecular dynamic simulation of the docked complex was performed using Desmond (D.E Shaw Research and Schrödinger, New York, NY, 2025).


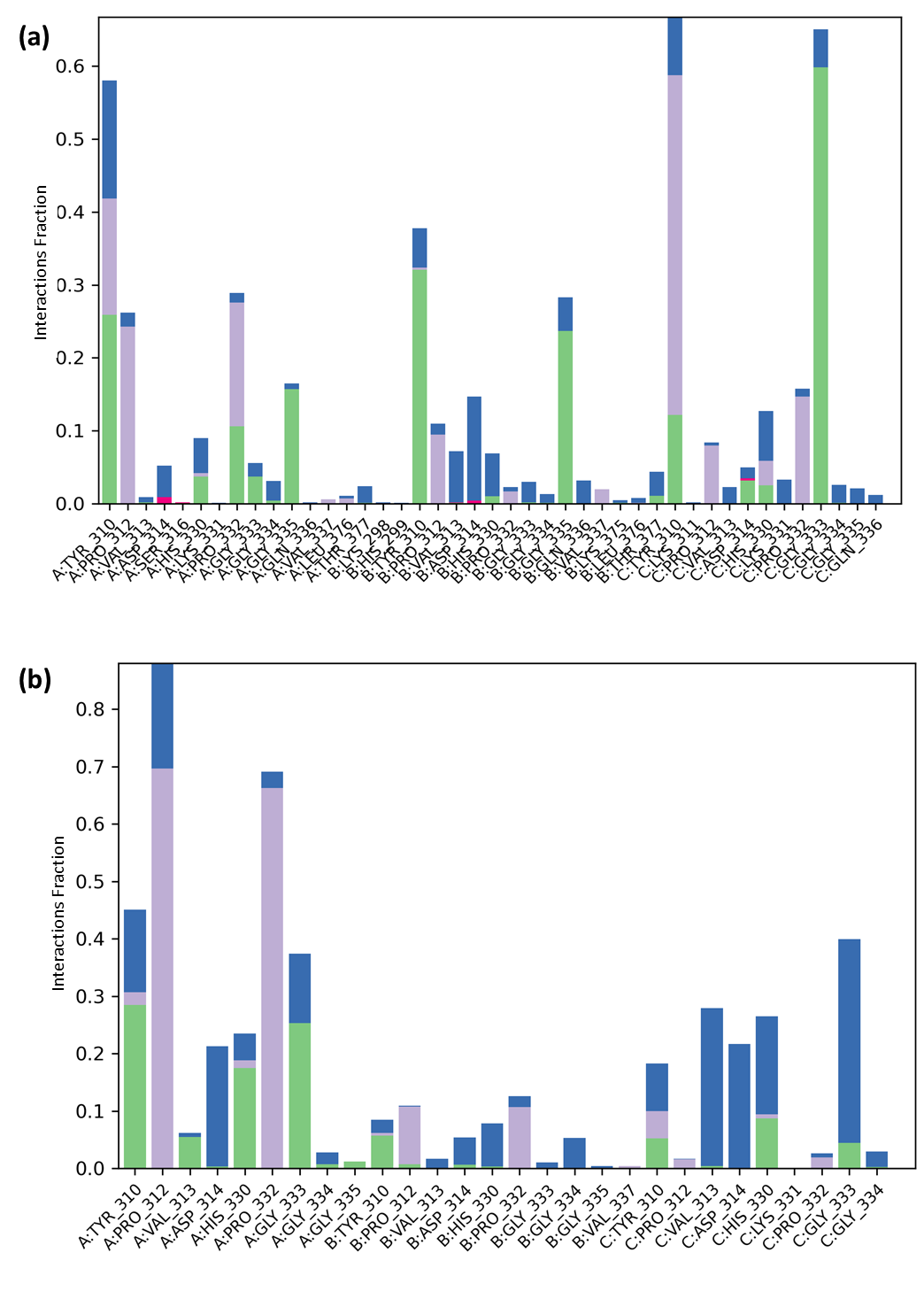


**Figure S15.** **Molecular simulation studies of P301L tau filament with compounds.** A schematic representation of the interaction of 9GG0 with the compounds (a) Oleocanthal and (b) Purpurin in molecular dynamics simulations. Interactions that occurred more than 30% of the simulation time in the selected trajectory (0.00 through 100 ns) are shown. These interactions can be categorized by the types of bonding, which are normalized over the course of the trajectory. Each interaction type contains more specific subtypes, which can be explored through the “Simulation Interaction Diagram” panel. These interactions (or “contacts”) are mainly categorized into three types and are represented in the above plot as hydrogen (green), hydrophobic (purple), and water bridge (blue) bonds. The docking-based interaction studies were carried out with the help of Schrödinger Release 2025-1. The molecular dynamic simulation of the docked complex was performed using Desmond (D.E Shaw Research and Schrödinger, New York, NY, 2025).

**
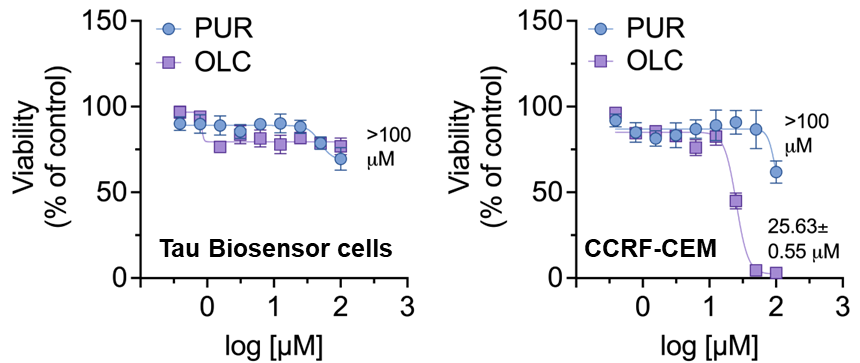
**

**Figure S16**. **Cytotoxic activity of PUR and OLC.** Tau Biosensor and CCRF-CEM (human leukemic lymphoblast) cells were treated with increasing concentrations (0-100 µM) of PUR and OLC for 72 h. Cell viability was then assessed using the MTS assay. CCRF-CEM (human leukemic lymphoblasts) cells, known for their high sensitivity to cytotoxic agents ^1^, were used as positive controls. Viability analysis revealed that neither PUR nor OLC was cytotoxic to Tau Biosensor cells within the tested concentration range. OLC exhibited cytotoxicity against CCRF-CEM cells, although with a half-maximal inhibitory concentration (IC_50_) exceeding 10 µM. Calculated IC_50_ values are indicated in the graphs. Mean ± SEM (*n* = 3).

Reference:

1. Medh RD, Webb MS, Miller AL, Johnson BH, Fofanov Y, Li T, Wood TG, Luxon BA, Thompson EB. Gene expression profile of human lymphoid CEM cells sensitive and resistant to glucocorticoid-evoked apoptosis. Genomics 2003;81(6):543–555


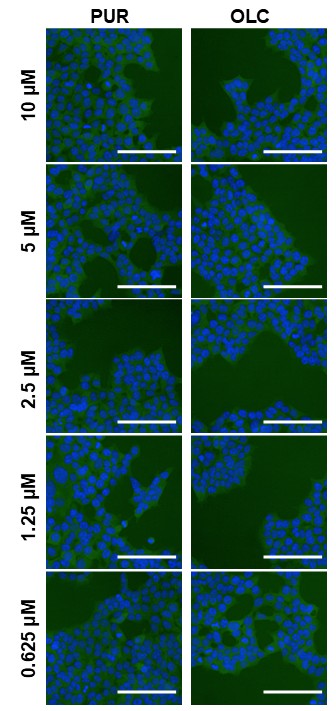


**Figure S17**. **PUR and OLC do not induce tau aggregation in the absence of exogenous seeds**. Tau RD P301S FRET Biosensor cells were treated with PUR or OLC at the indicated concentrations for 48 h in the absence of any exogenous tau seeds or aggregation mixtures. Cells were then imaged under the same conditions described in Figure 5 to assess spontaneous FRET signal (CFP/YFP inclusions) as a readout of intracellular tau aggregation. No FRET-positive aggregates were detected in any treatment condition, indicating that neither PUR nor OLC induces endogenous tau seeding activity. Scale bar: 100 µm.


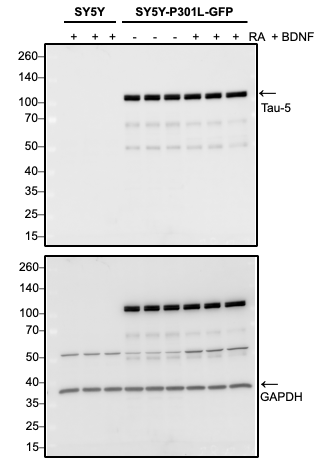


**Figure S18**. **Western blot validation of high molecular weight Tau-5 bands in SY5Y-TauP301L cells.** Western blot analysis of Tau-5 and GAPDH in lysates from differentiated SY5Y parental cells and undifferentiated and differentiated SY5Y-TauP301L cells. High molecular weight Tau-5 bands (arrows) are detected only in SY5Y-TauP301L cells and are absent in parental SY5Y cells, confirming their origin from the transfected plasmid. GAPDH was used as a loading control. Each condition includes three lanes, representing 3 independent replicates.


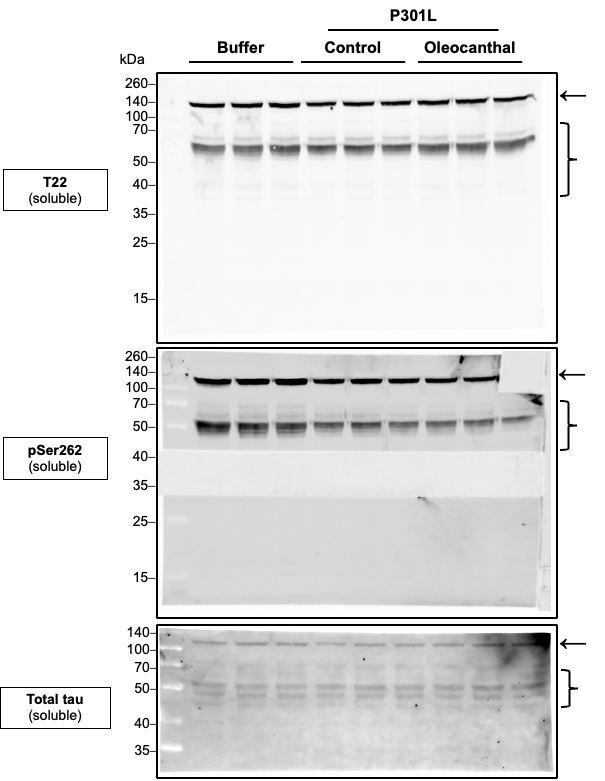


**Figure S19. Soluble tau analysis from SY5Y-P301L cells.** Western blot analysis of soluble fractions from SY5Y-TauP301L cells transduced with either buffer (reaction mixture without aggregation products) or P301L tau aggregation products formed in the absence (control) or presence of oleocanthal. The blots were probed for pSer262 phosphorylated tau, T22 (oligomeric tau), and Total tau. Arrows indicate the position of Tau(P301L) 2N4R EGFP expressed from the transfected plasmid, whereas brackets denote endogenous SY5Y tau.


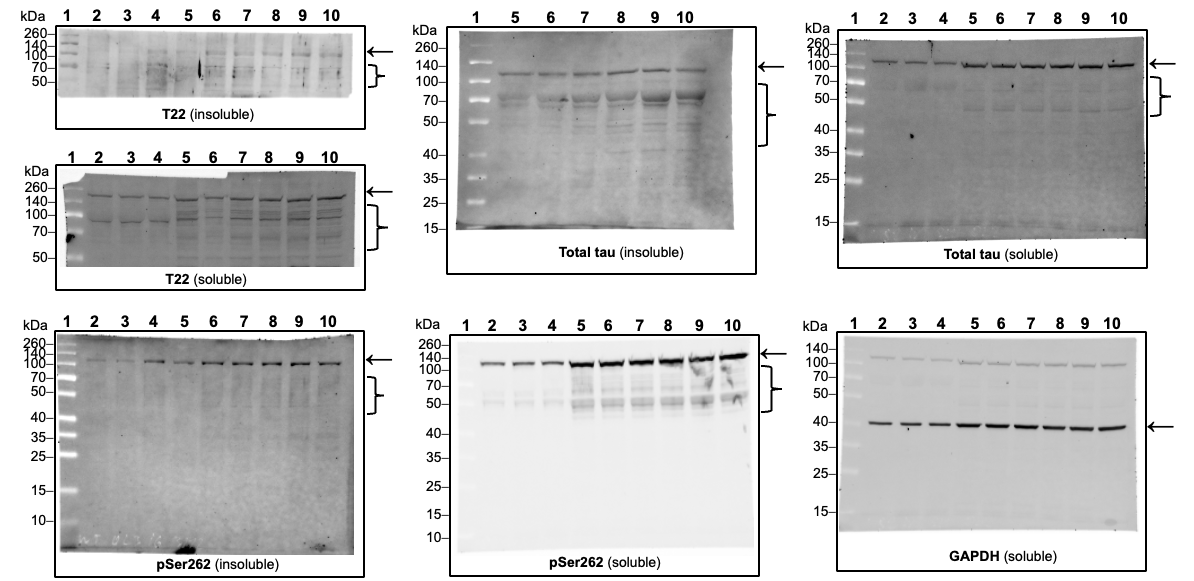


**Figure S20. Western blot analysis of SY5Y-TauP301L cells transduced with WT tau aggregation products.** Western blot analysis of insoluble and soluble fractions from SY5Y-TauP301L cells transduced with WT tau aggregation products formed in the absence (control) or presence of oleocanthal. Blots were probed for T22 (oligomeric tau), pSer262 (phosphorylated tau), total tau, and GAPDH (loading control from soluble fraction). Arrows indicate the position of Tau(P301L)2N4R EGFP expressed from the transfected plasmid, whereas brackets denote endogenous SY5Y tau. Lane 1: Molecular weight marker; Lanes 2-4: Buffer only control (reaction mixture without aggregation products); Lanes 5-7: WT tau aggregation products formed in the absence of oleocanthal (control); Lanes 8-10: WT tau aggregation products formed in the presence of oleocanthal.


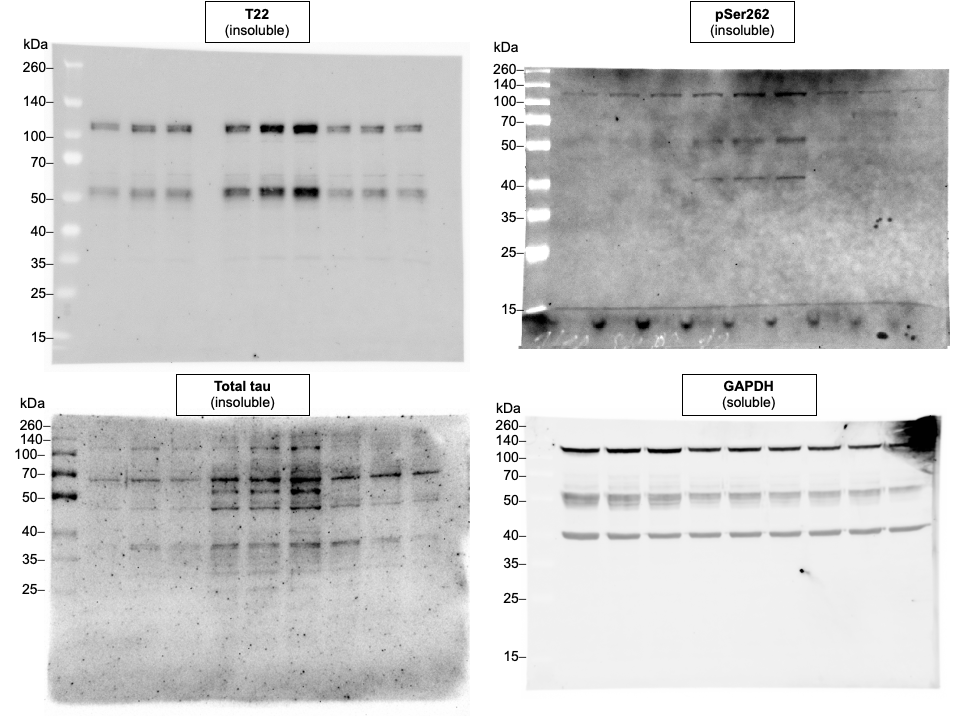


**Figure S21**. **Full-length Western blot images for T22, pSer262, total Tau, and GAPDH from Figure 8.** Western blot analyses were performed on insoluble fractions from SY5Y-TauP301L cells transduced with P301L tau aggregation products formed in the presence or absence of oleocanthal. Blots were probed for T22 (oligomeric tau), pSer262 phosphorylated tau, and Total tau. GAPDH from the soluble fraction was used as a loading control to confirm equal protein loading across samples. Each condition includes three lanes, representing 3 independent replicates.

**Table S1.** **Induced Fit Docking (IFD) scores for OLC and PUR with AlphaFold2-predicted WT and P301L tau peptide models**. The table summarizes docking results generated using the MOE 2024.0601 platform, including S-scores, ligand conformational energies (E_conf), placement scores (E_place), and refined interaction energies (E_refine) for each ligand-protein complex. RMSD values (Rmsd_refine) represent conformational deviations after refinement. Lower (more negative) energy values indicate more favorable binding interactions.

| **Ligand** | **Peptide Model** | **S-score** | **Rmsd_refine** | **E_conf** | **E_place** | **E_score1** | **E_refine** | **E_score2** |
| --- | --- | --- | --- | --- | --- | --- | --- | --- |
| OLC | WT | -4.45 | 1.75 | -34.63 | -40.20 | -6.76 | -19.53 | -4.45 |
| PUR | WT | -4.06 | 4.37 | 3.42 | -21.15 | -6.18 | -18.38 | -4.06 |
| OLC | P301L | -4.72 | 1.20 | -34.74 | -48.78 | -7.35 | -20.13 | -4.72 |
| PUR | P301L | -4.12 | 1.22 | 3.00 | -30.90 | -8.57 | -19.98 | -4.12 |

**Table S2.** **MM-GBSA binding free energy calculations for OLC and PUR docked to AlphaFold2-predicted WT and P301L tau peptide models**. Binding free energies (ΔG_bind) and their contributing energy components, including Coulombic, covalent, hydrogen bonding, lipophilic, solvation (GB), and van der Waals interactions, were calculated using the Prime MM-GBSA method in Schrödinger Maestro 2025-1. More negative ΔG_bind values reflect stronger predicted ligand-protein binding affinities.

| **Ligand** | **Peptide Model** | **ΔG Bind** | **ΔG Bind_Coulomb** | **ΔG Bind_Covalent** | **ΔG Bind_H bond** | **ΔG Bind_Lipophilic** | **ΔG Bind_Solvation_GB** | **ΔG Bind_vdW** |
| --- | --- | --- | --- | --- | --- | --- | --- | --- |
| OLC | WT | -27.89 | -12.83 | 0.99 | -0.73 | -6.91 | 13.38 | -21.64 |
| PUR | WT | -16.79 | -12.68 | 0.89 | -0.41 | -4.8 | 17.18 | -15.97 |
| OLC | P301L | -37.31 | -11.72 | 0.6 | -0.67 | -8.19 | 13.98 | -31.03 |
| PUR | P301L | -23.07 | -26.51 | 0.7 | -0.96 | -5.67 | 27.02 | -17.53 |
